# A hybrid analytical–optimization framework for sidelobe suppression and beamwidth control in linear antenna arrays

**DOI:** 10.1038/s41598-026-46772-8

**Published:** 2026-04-13

**Authors:** Ahmed M. Elkhawaga, Mohamed Aboualalaa, Mustafa M. Abd Elnaby

**Affiliations:** 1 Electronics and Electrical Communication Engineering Department, Faculty of Engineering, Tanta University, Tanta, 31527 Egypt; 2 Microstrip Department, Electronics Research Institute (ERI), Cairo, 11843 Egypt

**Keywords:** Uniform linear antenna array (ULAA), Side lobe level (SLL), Dynamic range ratio (DRR), Genetic algorithms (GA), Engineering, Mathematics and computing

## Abstract

This paper presents a novel hybrid analytical–optimization framework for sidelobe suppression and beamwidth control in uniform linear antenna arrays (ULAAs). The primary objective is to achieve significant sidelobe level (SLL) reduction while maintaining a controlled half-power beamwidth (HPBW) through a computationally efficient synthesis strategy. The proposed Enhanced Window-Based Array Synthesis Algorithm (EWASA), also referred to as the Raised Cosine Synthesis with Genetic Algorithm (RCS-GA), is built upon two key innovations. The proposed approach introduces a deterministic spatial shaping mechanism derived from the raised cosine function, originally used in digital communication pulse shaping, and adapts it to the angular domain for radiation pattern control. Unlike conventional tapering techniques that rely heavily on iterative optimization, the desired array response is first constructed analytically using the raised cosine spatial mapping. A closed-form matrix inversion technique then computes the excitation coefficients required to synthesize this target pattern. To further enhance performance, a genetic algorithm optimizes the inter-element spacing, enabling improved sidelobe suppression while maintaining beam integrity. This hybrid approach significantly reduces the dimensionality of the optimization problem and accelerates convergence. Simulation results demonstrate substantial improvements, achieving an SLL of − 38.05 dB and an HPBW of 5.526° for a 15-element array—representing a threefold reduction in SLL and more than 50% improvement in HPBW compared to conventional uniform arrays. The proposed technique maintains a practical excitation dynamic range, and full-wave CST Microwave Studio simulations confirm its practical feasibility. Owing to its computational efficiency, non-iterative core, and precise beam control capability, the proposed method is particularly suitable for high-resolution applications in radar systems, electronic warfare, and microwave medical imaging where interference suppression and beamforming accuracy are critical.

## Introduction

Antenna arrays play a crucial role in modern wireless communication, radar, and sensing systems due to their capability to achieve high directivity and flexible radiation pattern control. In many practical applications, such as phased-array radar, satellite communication systems, and microwave imaging, it is essential to design array configurations that simultaneously achieve narrow beamwidth and SLL. These requirements are often conflicting because reducing the sidelobe level typically results in an increase in the main-lobe width, making the synthesis of antenna arrays a challenging optimization problem.

Several classical techniques have been proposed in the literature to address this trade-off. One of the most widely used approaches relies on amplitude tapering, where the excitation coefficients of the array elements are adjusted using predefined window functions. Well-known examples include Chebyshev, Taylor, and raised cosine distributions, which are designed to control sidelobe behavior while maintaining acceptable beamwidth characteristics. Although these methods provide analytical solutions and predictable sidelobe performance, they often require non-uniform excitation amplitudes that may increase implementation complexity and reduce power efficiency.

Another class of methods focuses on non-uniform array synthesis, where the positions of the radiating elements are optimized instead of modifying their excitation amplitudes. By properly adjusting the inter-element spacing, it is possible to suppress sidelobes while maintaining uniform excitations, which simplifies the feeding network and improves hardware efficiency. However, determining optimal element positions generally leads to a highly nonlinear optimization problem. As a result, many recent studies have employed evolutionary optimization techniques, such as GA, particle swarm optimization (PSO), and differential evolution, to search for optimal array configurations.

High-resolution antenna array synthesis is crucial in advanced communication, radar, and medical imaging systems, where precise beam shaping and sidelobe suppression directly influence accuracy and system efficiency. For instance, in ultra-high-field (UHF) MRI systems and circular microwave imaging arrays, standard performance requirements typically include SLL below − 30 dB and HPBW ranging between $4^\circ$ and $6^\circ$ to ensure spatial selectivity and minimize diagnostic artifacts^1,2^.

Traditional approaches to array pattern control have often combined numerical electromagnetic analysis techniques with stochastic optimizers. For instance, the Method of Moments (MoM) has been used as an accurate analysis engine within hybrid frameworks, where it is coupled with Genetic Algorithms (GA) to iteratively optimize array parameters for desired radiation characteristics. However, these techniques often rely on iterative optimization and face limitations in convergence speed, complexity, and robustness, particularly when scaling to large or non-uniform arrays. Recent approaches have addressed these challenges through hybrid metaheuristics and algorithmic steering. For example, Amer et al. introduced hybrid convolution/genetic algorithm techniques for 5G and beyond communications, achieving significant SLL reduction and improved beamforming performance^3^. Similarly, Asaad and Hreshee applied GA, Flower Pollination Algorithm (FPA), and Grey Wolf Optimization (GWO) to linear antenna arrays, demonstrating effective SLL suppression across various array sizes^4^.

Beyond classical tapering and optimization-based synthesis techniques, alternative structural and robustness-oriented approaches have also been investigated to control sidelobe levels in antenna arrays. One prominent direction is the use of clustered subarray configurations, where groups of elements are combined to form subarrays whose collective excitation is optimized to shape the overall radiation pattern. Such approaches have been shown to provide effective sidelobe suppression in both linear and planar arrays while reducing the dimensionality of the optimization problem. For example, a T-shaped polyomino subarray design was proposed to achieve enhanced sidelobe control through geometrical clustering, demonstrating notable improvements in sidelobe level reduction without relying solely on amplitude tapering techniques^5^. In parallel, weight perturbation methods have been explored as a lightweight alternative to full re-optimization, where small controlled deviations are applied to the excitation weights to suppress sidelobes while preserving the main beam characteristics^6^.

In addition to synthesis strategies, robustness against practical imperfections remains a critical consideration in array design. Random errors in amplitude and phase excitations, arising from manufacturing tolerances, quantization effects, and feeding network inaccuracies, can significantly distort the synthesized radiation pattern and degrade sidelobe performance. Prior studies have analytically and numerically investigated the sensitivity of adaptive nulling and sidelobe suppression schemes to such random excitation errors, highlighting the necessity of synthesis methods that maintain acceptable performance under realistic perturbations^7^. These works collectively emphasize that effective array synthesis must balance sidelobe suppression capability, structural efficiency, and robustness to implementation errors. Motivated by these observations, the present work focuses on a deterministic analytical shaping framework combined with constrained optimization of element spacing, aiming to achieve deep sidelobe suppression with reduced sensitivity to excitation inaccuracies and without resorting to complex subarray clustering or purely iterative weight perturbation schemes.

In the realm of medical imaging, advancements have been made to enhance MRI performance at ultra-high fields. Egorova et al. developed a bore-integrated patch antenna array designed for whole-body excitation in UHF MRI, utilizing the MRI bore’s RF shield as a functional component to enhance the RF magnetic field distribution^8^. Additionally, Koloskov et al. evaluated the use of flexible meta-surfaces to improve brain imaging at 7T, leading to increased homogeneity of the transmit field and improved diagnostic image quality^9^. Alipour et al. proposed a meta-array structure to enhance brain MRI at ultra-high field systems, resulting in improved transmit efficiency and signal sensitivity^10^.

Further innovations include the development of a coupled planar transmit RF array for ultrahigh field spine MR imaging which demonstrated superior performance compared to conventional surface coils in terms of B1 efficiency and specific absorption rate (SAR)^11^. Bhosale et al. introduced discrete dielectric material-coated dipole antennas for UHF MRI applications, improving frequency tuning and reducing SAR^12^. Moreover, the use of 3D metamaterials has been proposed to facilitate human cardiac MRI at $21.0\;{\mathrm{Tesla}}$, enhancing ${B1}^{+}$ efficiency and uniformity across the human heart^13^.

In radar and microwave imaging applications, advancements have been made in beamforming techniques. Shi et al. presented a phase-only transmit beampattern synthesis method for cluttered environments in airborne radar, improving target detection capabilities^14^. Liu and Pan researched beampattern synthesis of conformal arrays based on space-variability suppression, enhancing beam-forming accuracy^15^. Lan et al. proposed a beampattern synthesis and optimization method based on a circular frequency diverse array engineering model, offering improved main-lobe interference mitigation^16^.

Additionally, Alvarez López and Las-Heras Andrés improved methods for Fourier-based microwave imaging, enhancing image reconstruction quality^17^. Qiu et al. reviewed advances and prospects in SAR microwave vision three-dimensional imaging, highlighting the integration of artificial intelligence in radar imaging^18^.

A wide spectrum of recent studies has explored different optimization and synthesis perspectives. Time-modulated array architectures have been used for harmonic and sidelobe suppression by employing pulse-splitting techniques^19^. Convex-optimization-based element perturbation has also been shown effective for precise sidelobe regulation without resorting to heavy stochastic computations^20^. Genetic-algorithm-based optimization continues to attract interest due to its flexibility, as seen in recent designs of low-SLL dipole arrays and non-uniform arrays^21^. Other contemporary contributions include enhanced Harris Hawks Optimization (HHO) for suppressing peak sidelobe levels in spiral arrays^22^, time-modulated arrays with ultralow SLL^23^, and deep-learning-assisted sparse array design that accelerates pattern synthesis beyond conventional iterative methods^24^. More recently, polarization-coded reconfigurable phased arrays have been introduced to simultaneously address wideband synthesis and polarization diversity^25^.

On another front, significant advances have been made in applied imaging systems—particularly microwave medical imaging, where strict SLL and HPBW requirements determine achievable spatial resolution and artifact suppression. For example, modern microwave imaging platforms typically require sidelobe levels below − 30 dB and HPBW values within 3°–5° to meet clinical resolution benchmarks^26^, motivating the need for deterministic and computationally efficient pattern-shaping methods.

Despite recent advancements, there remains a critical gap in establishing analytically tractable excitation strategies that simultaneously offer precise beam shaping and rigorous electromagnetic field accuracy. To address this, the present work introduces a novel hybrid framework that integrates the Raised Cosine (RC) shaping function with an inverse matrix technique, effectively bridging concepts from digital pulse shaping and antenna array theory. In this approach, the RC function serves as the target spatial excitation profile, enabling a direct and systematic translation of time-domain shaping principles into the angular domain. This eliminates the need for extensive iterative optimization and facilitates high-precision control over radiation characteristics.

The novelty of this paper is therefore not the raised-cosine function itself, which is indeed a conventional window, but its novel adaptation and the resulting synthesis framework. The proposed method introduces a paradigm shift by:

1. Establishing an analytical equivalence between the time-domain RC pulse-shaping variable and the spatial angular frequency of the array factor, allowing the complete RC function to serve as the target radiation pattern.
2. Developing a closed-form, matrix-inversion-based synthesis technique to solve for the excitation coefficients that inherently produce this RC-defined pattern, moving beyond simple multiplicative tapering.
3. Integrating this deterministic shaping with a genetic algorithm for element spacing optimization, creating a hybrid RCS-GA framework that is largely non-iterative for excitation calculation.

This approach bridges concepts from digital communication theory and antenna array synthesis into a unified, analytically tractable design procedure, offering a significant advantage in computational efficiency and precision over fully iterative optimization methods.

In the proposed framework, the parameter $\beta$ represents the roll-off factor of the raised cosine function used to construct the desired spatial radiation pattern. This parameter controls the smoothness of the transition between the main lobe and the sidelobe region of the synthesized pattern. By adjusting the value of $\beta$, the algorithm can regulate the trade-off between SLL suppression and main-lobe beamwidth. Larger values of $\beta$ produce smoother spatial tapering, which typically results in stronger sidelobe attenuation but slightly broader beamwidth. In contrast, smaller values lead to sharper transitions that preserve beamwidth at the expense of higher sidelobe levels. Consequently, $\beta$ serves as a pattern-shaping control parameter that allows flexible adjustment of the desired radiation characteristics prior to the optimization stage.

It is important to note that the raised cosine function itself is not introduced as a new tapering function in this work. Instead, the novelty of the proposed method lies in the way this function is utilized within a hybrid synthesis framework for antenna array pattern design. Specifically, the raised cosine formulation is employed to analytically construct a desired spatial radiation pattern, which serves as a deterministic target for the synthesis process. The excitation coefficients required to approximate this pattern are then obtained through a closed-form matrix inversion technique, while a genetic algorithm is used to optimize the inter-element spacing. This combination of analytical pattern construction and geometry optimization significantly reduces the dimensionality of the optimization problem compared with conventional array synthesis techniques that simultaneously optimize amplitudes and element positions.

The novelty of this work lies not in the use of the raised cosine function itself—which has been previously utilized in signal processing and array tapering—but in the proposed hybrid synthesis framework that integrates analytical spatial pattern construction with optimization-based array geometry design. Unlike conventional antenna array synthesis techniques that rely primarily on amplitude tapering or fully stochastic optimization of excitation parameters, the proposed approach first constructs a deterministic target radiation pattern analytically in the spatial domain. The excitation coefficients required to synthesize this pattern are then obtained through a closed-form matrix inversion formulation, eliminating the need for iterative amplitude optimization. Subsequently, a genetic algorithm is employed exclusively to optimize the inter-element spacing, enabling further sidelobe suppression while preserving beamwidth characteristics. This decomposition significantly reduces the dimensionality of the optimization problem, leading to improved convergence behavior and reduced computational complexity compared with traditional array synthesis approaches. Consequently, the proposed framework provides an efficient methodology for achieving simultaneous sidelobe level reduction and beamwidth control while maintaining practical excitation dynamic range, making it particularly suitable for high-resolution antenna array applications.

Key contributions of Enhanced Window-Based Array Synthesis Algorithm (EWASA) include:

1. Superior SLL Reduction with Favorable Beamwidth: EWASA achieves significant SLL suppression that surpasses conventional tapering techniques. For a $15 -$ element array with β = 1, the proposed method achieves an SLL of $- 38.05\;{\mathrm{dB}}$—a threefold reduction compared to the uniform array ($- 13.2\;{\mathrm{dB}}$). This represents an improvement of $8 - 10\;{\mathrm{dB}}$ over standard Chebyshev and Taylor windows designed for − 30 dB SLL , and 3–5 dB over optimized Kaiser window designs . Crucially, this enhanced SLL suppression is accompanied by a narrower HPBW $\left( {5.53^\circ } \right)$ compared to classical tapers $\left( {6.0 - 8.5^\circ } \right)$, demonstrating that EWASA the conventional performance trade-off limitations of amplitude-only tapering methods.
2. Adaptive HPBW Control: The proposed algorithm enables dynamic control of the HPBW through variation in $\beta$ variations. For example, when $\beta = 1$, the resulting HPBW is $5.526^\circ$ with an SLL of $- 38.05\;{\mathrm{dB}}$, whereas for $\beta = 2$, the HPBW narrows to $4.806^\circ$ with a corresponding SLL of $- 29.05\;{\mathrm{dB}}$.
3. First-Sidelobe Level (FSLL) Management: The algorithm ensured effectively suppresses the FSLL, with values reaching $- 42.8\;{\mathrm{dB}}$ for $\beta = 1$ and $- 30.16\;{\mathrm{dB}}$ for $\beta = 2$.
4. Scalability and Practical Validation: EWASA is validated through extensive numerical simulations and full-wave electromagnetic analysis in CST Microwave Studio, confirming its applicability in real-world phased array systems.
5. Potential Applications: The proposed method is highly beneficial for EW, radar systems, and medical imaging applications, where precise beam shaping and interference mitigation are crucial.

This study presents a novel and efficient inverse matrix and GA-based optimization framework for SLL reduction in uniform linear antenna arrays (ULAAs). The proposed methodology is distinct from existing techniques in several key aspects:

I. Integration of a Custom-Designed Shaping Function

Unlike conventional approaches that rely on iterative optimization or predefined windowing functions, this study introduces a custom-engineered shaping function that directly influences the array factor $AF\left(\theta \right)$ This function strategically maintained the integrity of the main beam while suppressing undesired sidelobes.

II. Optimized Element Spacing and Excitation Coefficients

The study eliminates the need for uniform element spacing by optimizing the inter-element distances and excitation coefficients using the inverse matrix framework. This approach ensures a more flexible and efficient sidelobe suppression mechanism, thereby improving overall radiation performance.

III. Two-Step Synthesis Approach for Maximum SLL Reduction

A well-structured, two-step synthesis process is developed:

- *Step 1*: The shaping function is applied to generate the desired array pattern ${AF}_{d}\left(\theta \right)$, ensuring predefined beam characteristics.
- *Step 2*: The inverse matrix technique is leveraged to synthesize the array by determining the optimal excitation coefficients and element spacing, achieving superior SLL reduction.

IV. Superior Trade-off Between Beamwidth and Sidelobe Control

The proposed method effectively balanced HPBW reduction and sidelobe suppression without excessive computational complexity, making it highly suitable for practical implementations.

V. Significant Performance Gains Over State-of-the-Art Methods

Comparative analysis demonstrated that the proposed method outperformed traditional optimization-based techniques such as Iterative Fourier Transform (IFT), Differential Evolution (DE), and Threshold Sparse IFT (TS-IFT).

1. SLL suppression: The proposed method achieves an *SLL* of $-38.05$ dB compared to $-13.1$ dB in conventional ULAAs.
2. Narrower beamwidth: improving directivity while maintaining controlled sidelobe growth.
3. FSLL reduction: enhancing overall radiation pattern efficiency.

The full-wave array performance is validated through CST Microwave Studio simulations, demonstrating an SLL of $-38.05$ dB and HPBW of $5.526^\circ$, surpassing the performance of standard rectangular and Chebyshev tapering windows. These results meet beam quality requirements for bore-integrated MRI arrays, circular MIMO imaging systems, and electronically reconfigurable reflector systems.

A core innovation of this work lies in the analytical demonstration that the raised cosine (RC) function can be decomposed into two conceptual components to enable precise shaping of the array radiation pattern. The first component corresponds to a shaping function, mathematically derived from part of the RC envelope, which acts as an angular weighting applied to the classical uniform linear array factor. This shaping function governs the sidelobe roll-off and main-lobe smoothness.

The second, and arguably more fundamental, component pertains to the formulation of the uniform linear array factor (ULAA) itself. The key novelty lies in establishing that the complete raised cosine (RC) function—rather than being a mere shaping filter—constitutes the desired array response in its entirety. This means that the RC function is not applied externally as a post-synthesis weight but instead serves as the target radiation pattern that the array factor must inherently follow. Consequently, the synthesis process involves solving for the excitation current vector such that the ULAA matrix model directly reproduces the full RC behavior. This is achieved through a closed-form matrix inversion, ensuring that the resulting pattern faithfully approximates the entire RC envelope, rather than relying on heuristic tapering or localized sampling. This insight bridges the shaping objective and excitation synthesis into a unified, analytically tractable framework.

This two-part decomposition (1) shaping through partial RC projection and (2) synthesis of the full RC as a matrix target establishes a closed-form, non-iterative framework for antenna array pattern control. It also bridges the gap between time-domain pulse shaping theory and spatial-domain beam synthesis, enabling deterministic design with strong theoretical guarantees.

This paper proposes an efficient algorithm named Raised Cosine Synthesis with Genetic Optimization for Linear Antenna Arrays )RCS-GA), which integrates raised cosine spatial shaping, closed-form matrix inversion, and a genetic optimization framework for element spacing.

The remainder of this paper is organized as follows: “Problem definition” Section defines the problem formulation in the context of array pattern shaping and performance trade-offs. “Proposed SLL reduction and narrow HPBW of ULAA using RCS-GA” Section introduces the proposed SLL reduction and HPBW narrowing technique for ULAAs using the RCS-GA method. “Simulation results of proposed technique” Section presents detailed simulation results validating the effectiveness of the proposed approach. “CST simulations for practical validation” Section provides practical validation using CST-based full-wave electromagnetic simulations. “Comparison with related work” Section offers a comparative analysis with related state-of-the-art techniques in terms of performance and computational efficiency.

## Problem definition

The problem addressed in this study centers on the analytical formulation and accurate realization of array excitation profiles that ensure both optimal sidelobe suppression and precise beam control. For a uniform linear antenna array (ULAA) composed of $N$ isotropic elements equally spaced along the x-axis, the array factor (AF) can be expressed as^27^:

1 $$AF_{ULAA} \left( \theta \right) = \mathop \sum \limits_{n = 1}^{N} w_{n} e^{{jk\left( {n - 1} \right)d{\mathrm{cos}}\left( \theta \right)}}$$

Here, ${w}_{n}$ denotes the excitation coefficient of the *n*th element, $k=2\pi /\lambda$ is the wave number, $d$ is the inter-element spacing, and $\theta$ is the angle of observation.

The excitation coefficients ${w}_{n}$ in Eq. (1) are complex-valued quantities representing the excitation amplitude and phase applied to the *n*th antenna element. These coefficients determine the contribution of each element to the overall array radiation pattern.

In the initial formulation, the array is assumed to be a uniform linear antenna array (ULA) in which the elements are equally spaced along the array axis. The array is oriented along the z-axis, and the observation direction is defined by the spherical coordinate angle $\theta$, measured from the array axis.

Under these assumptions, the array factor can be expressed as shown in Eq. (1). For the special case of uniform excitation, the amplitude coefficients are normalized such that ${w}_{n}=1$

for all array elements. In this case, the excitation coefficients are identical and have zero phase difference, resulting in maximum radiation in the broadside direction.

It should be noted that although the initial formulation assumes equispaced elements, the proposed synthesis method later introduces inter-element spacing optimization, allowing the array geometry to deviate from uniform spacing in order to improve sidelobe suppression performance.

**Fig. 1:**
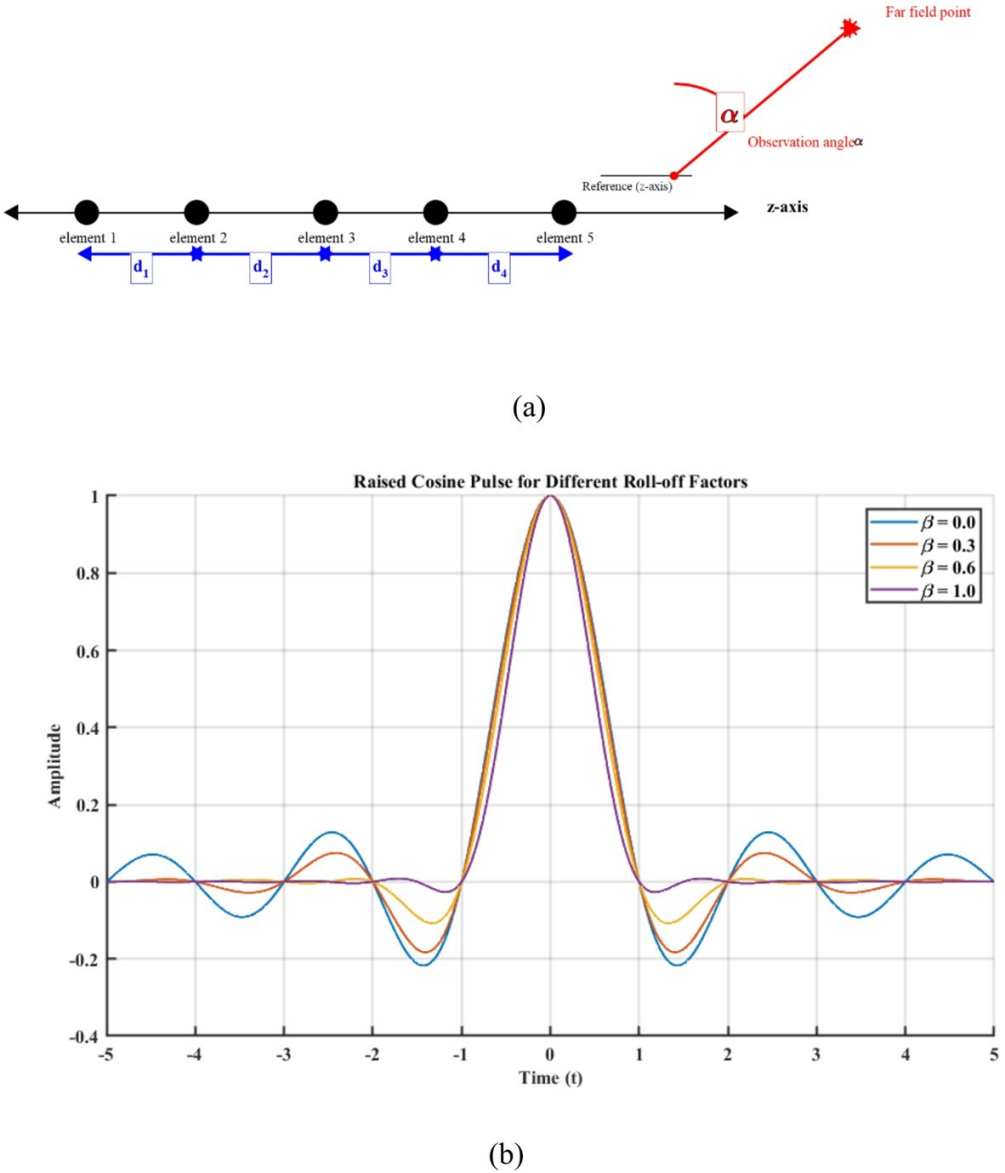
(**a**) Geometry of the linear antenna array showing the array axis, inter-element spacing, and observation angle definition used in Eq. (1), (**b**) Raised Cosine pulse shaping.

A schematic representation of the array geometry and the definition of the observation angle are illustrated in Fig. 1a.

For a ULAA with symmetric element placement and uniform excitation amplitudes ($w_{n} = 1$), the resulting pattern for a large number of elements $N$ approximates a $\sin c$ function, with sidelobe levels that must be carefully shaped for practical applications. The shaping function selected in this work is the RC function, chosen for its smooth roll-off and superior sidelobe attenuation properties.

### Definition of raised cosine function

The RC function is a well-established tool in digital communication systems, primarily utilized for pulse shaping to mitigate inter-symbol interference (ISI). Its well-known properties are briefly reviewed here as a foundation for its novel application to spatial beamforming, which is the core contribution of this work. The subsequent subsections will detail the original mapping and synthesis framework that leverages this function.

The RC pulse plays a fundamental role in digital communication by ensuring smooth spectral characteristics and minimizing ISI. The roll-off factor $\beta$ provides a trade-off between bandwidth efficiency and time-domain compactness. The MATLAB implementation illustrates how different $\beta$ values affect pulse shape as shown in Fig. 1b, highlighting the significance of choosing an optimal $\beta$ for a given communication system.

Mathematically, the time-domain representation of the Raised Cosine pulse is given by^28^:

2 $$p\left( t \right) = \frac{{\sin \left( {\frac{\pi t}{T}} \right)}}{{\frac{\pi t}{T}}} \cdot \frac{{\cos \left( {\frac{\pi \beta t}{T}} \right)}}{{1 - \left( {\frac{2\beta t}{T}} \right)^{2} }}$$

where $T$ is the symbol duration, and $\beta$ is the roll-off factor that controls the excess bandwidth of the pulse. The key parameters influencing the shape and performance of the Raised Cosine function include:

- Symbol Duration (T): Defines the temporal width of the pulse and directly impacts the bandwidth.
- Roll-off Factor ($\beta$): Determines the excess bandwidth beyond the Nyquist rate. It ranges from 0 to 1, where $\beta =0$ corresponds to an ideal sinc pulse, and $\beta =1$ leads to the widest transition band.
- Time (t): Represents the independent time variable over which the function is evaluated.

### Rationale for selecting the raised cosine function

The relationship $\frac{\pi t}{T} = \frac{N}{2}kd\cos \left( \theta \right)$ establishes a fundamental equivalence between a time-domain parameter and a spatial angular frequency in linear antenna arrays. This mapping underpins the adaptation of shaping functions, such as the RC, from temporal pulse design in digital communications to spatial beamforming applications in antenna synthesis.

*Step 1*: Coordinate Transformation—From Cartesian to Spherical Domain

Consider a 1D ULAA aligned along the x-axis. ${x}_{n}=n\cdot d$ is the position of the *n*th element.

When a plane wave arrives at angle $\theta$, its projection onto the array axis becomes:

3 $$r_{n} = x_{n} \cos \left( \theta \right) = nd\cos \left( \theta \right)$$

This leads to a phase shift at each element due to the wavefront:

4 $$\phi_{n} = kx_{n} \cos \left( \theta \right) = knd\cos \left( \theta \right)$$

Hence, the array factor can be expressed as:

5 $$AF\left( \theta \right) = \mathop \sum \limits_{n = 0}^{N - 1} w_{n} e^{jknd\cos \left( \theta \right)} \cong \frac{{\sin \left( {\frac{N}{2}kd\cos \left( \theta \right) } \right)}}{{\frac{N}{2}kd\cos \left( \theta \right) }}$$

This is a $sinc$-like response representing the baseline uniform array beam-pattern.

*Step 2*: Temporal Raised Cosine Function

The classical raised cosine pulse in time-domain communications is defined as:

6 $$h\left( t \right) = \frac{{\sin \left( {\frac{\pi t}{T}} \right)}}{{\frac{\pi t}{T}}}\frac{{\cos \left( {\pi \beta t/T} \right)}}{{1 - (2\beta t/T)^{2} }}$$

Here, the term $\frac{\pi t}{T}$ serves as the normalized shaping argument controlling the pulse’s spectral roll-off. In antenna array synthesis, the goal is to replicate such shaping in the angular domain using $\theta$ as the spatial variable.

To that end, the spatial shaping function is defined analogously as:

7 $$F_{{{\mathrm{shaping}}}} \left( \theta \right) = \frac{{\cos \left( {\beta x} \right)}}{{1 - \left( {\frac{2\beta }{\pi }x} \right)^{2} }}$$

where $x$ represents the spatial equivalent of the time-domain shaping argument.

*Step 3*: Mapping Time-Domain to Angular Domain

To establish the equivalence, we equate the normalized arguments in both domains:

8 $$\frac{\pi t}{T} \equiv x = \frac{N}{2}kd\cos \left( \theta \right)$$

The factor $\frac{N}{2}$ arises from modeling the array as symmetric about its center, spanning from $- \frac{N}{2}d$ to $+ \frac{N}{2}d$. The maximum phase deviation occurs at the outermost elements, yielding:$x_{\max } = \frac{N}{2}kd\cos \left( \theta \right)$.

This spatial term now acts as a direct analogue to the temporal shaping variable.

*Step 4*: Final Mapping and Interpretation

Thus, we establish the mapping:

9 $$\frac{\pi t}{T} \leftrightarrow \frac{N}{2}kd\cos \left( \theta \right)$$

This proves that time-domain shaping functions, such as the RC filter used in digital modulation, can be directly transformed into spatial shaping functions for antenna arrays using angular-domain coordinates.

By applying this concept, the shaped array factor becomes:

10 $$AF_{RC} \left( \theta \right) = AF_{ULAA} \left( \theta \right) \cdot F_{shaping} \left( \theta \right) = \underbrace {{\left[ {\frac{{\sin \left( {\frac{N}{2}kd\cos \left( \theta \right)} \right)}}{{\frac{N kd\cos \left( \theta \right)}{2}}}} \right]}}_{{AF_{ULAA} \left( \theta \right)}} \cdot \underbrace {{\frac{{\cos \left( {\frac{\beta Nkd\cos \left( \theta \right)}{2}} \right)}}{{1 - \left( {\frac{2\beta }{\pi }\frac{Nkd\cos \left( \theta \right)}{2}} \right)^{2} }}}}_{{F_{shaping} \left( \theta \right)}}$$

where ${F}_{shaping}(\theta )$ is the shaping function given by:

11 $$F_{shaping} \left( \theta \right) = \frac{{\cos \left( {\frac{\beta Nkd\cos \left( \theta \right)}{2}} \right)}}{{1 - \left( {\frac{2\beta }{\pi } \frac{Nkd\cos \left( \theta \right)}{2}} \right)^{2} }}$$

This formulation creates a mathematically rigorous bridge between digital signal shaping in time and radiation pattern shaping in space. It allows raised cosine filtering—widely used for spectral efficiency in communications—to be reinterpreted as a spatial tapering mechanism that enables narrow beamwidth and deep sidelobe suppression in antenna array synthesis.

In antenna array synthesis, sidelobe level (SLL) control is traditionally achieved through two primary mechanisms: excitation amplitude tapering and modification of the inter-element spacing. Amplitude tapering adjusts the excitation coefficients applied to each array element in order to suppress sidelobes, while non-uniform spacing modifies the array geometry to redistribute the radiated energy in the angular domain.

The proposed approach integrates these two concepts within a hybrid synthesis framework. First, a deterministic spatial shaping function is used to construct the desired radiation pattern with controlled sidelobe characteristics. Based on this analytically defined target response, the excitation coefficients are obtained through a closed-form matrix inversion formulation. This step determines the amplitude distribution required to approximate the desired pattern.

To further enhance sidelobe suppression, a genetic algorithm is subsequently employed to optimize the inter-element spacing, allowing the array geometry to deviate from uniform spacing. By combining analytical excitation synthesis with spacing optimization, the proposed method achieves improved sidelobe control while maintaining the desired beamwidth characteristics.

## Proposed SLL reduction and narrow HPBW of ULAA using RCS-GA

This section presents the proposed synthesis framework for SLL suppression and beamwidth control in ULAAs. Unlike conventional approaches that rely exclusively on amplitude tapering or full numerical optimization, the proposed method introduces a hybrid analytical–optimization framework that combines deterministic spatial pattern construction with evolutionary geometry optimization.

The key idea of the proposed approach is to decouple radiation pattern shaping from array geometry optimization. First, a desired radiation pattern is analytically constructed using a deterministic spatial shaping function. Then, the excitation coefficients required to reproduce this pattern are computed using a closed-form matrix formulation. Finally, the inter-element spacing is optimized using a GA in order to achieve improved sidelobe suppression while maintaining the desired beamwidth.

This strategy significantly reduces the dimensionality of the optimization problem compared with conventional array synthesis techniques where both excitation amplitudes and element positions are optimized simultaneously.

To ensure that the main beam remains intact while imposing controlled nulls in the remaining radiation pattern, an appropriately designed shaping function is applied to the original array factor $AF\left(\theta \right)$. This function effectively modifies the excitation distribution, guiding the pattern toward optimal sidelobe suppression. Once the main beam is distinctly preserved, the RCS-GA framework is employed to determine the optimal element spacing and excitation coefficients required for achieving maximum SLL reduction.

The synthesis process follows a structured two-step approach. The first step involves generating the desired array pattern ${AF}_{d}\left(\theta \right)$, which serves as a reference for shaping the radiation characteristics. The second step utilizes the RCS-GA technique to compute the synthesized excitation coefficients and inter-element spacing, ensuring a well-optimized trade-off between sidelobe suppression and beam fidelity.

To synthesize the excitation coefficients that generate the desired array factor pattern, we first express the problem in matrix–vector notation. Let the excitation vector be defined as:

12 $$\mathbf{W}=[{w}_{1},{w}_{2},\dots ,{w}_{N}{]}^{T}$$

and let the steering vector corresponding to an observation angle $\theta$ be given by:

13 $$\mathbf{A}(\theta )=[1,{e}^{jkd\mathrm{cos}(\theta )},{e}^{2jkd\mathrm{cos}(\theta )},\dots ,{e}^{(N-1)jkd\mathrm{cos}(\theta )}{]}^{T}$$

The desired array factor at angle $\theta$ is thus expressed as:

14 $$A{F}_{desired}(\theta )={\mathbf{W}}^{T}\cdot \mathbf{A}(\theta )$$

To discretize the synthesis problem, the desired pattern is sampled at $M$ discrete angles ${\theta }_{m}$, with $m=\mathrm{1,2},\dots ,M$. These samples define the desired response vector:

15 $${{\mathbf{W}}_{1\times N}.\mathbf{A}}_{N\times M}={{\mathbf{A}\mathbf{F}}_{d}}_{1\times M}$$

where $\mathbf{A}$ is the steering matrix and ${\mathbf{A}\mathbf{F}}_{d}$ is the vector of desired RC-shaped values.

Once the desired array factor ${AF}_{d}\left(\theta \right)$ is generated analytically based on the raised cosine profile, it becomes necessary to solve the system in Eq. (15) to determine the corresponding excitation coefficients and optimal element spacings. To facilitate efficient computation and enable closed-form analysis, Eq. (15) is reformulated into a compact matrix form. This transformation leverages discretization over a finite number of angular samples.

Let M denote the total number of angular sample points required to accurately represent the desired radiation pattern. The sampled version of the desired array factor is then expressed as:

16 $$\left[ {AF_{d} \left( \theta \right)} \right]_{1 \times M} = \left[ {AF_{d} \left( {\theta_{1} } \right) AF_{d} \left( {\theta_{2} } \right) AF_{d} \left( {\theta_{3} } \right) \ldots AF_{d} \left( {\theta_{M} } \right)} \right]$$

with sample angles defined by the vector: $\theta =\left[{\theta }_{1},{\theta }_{2},{\theta }_{3},\dots \dots ,{\theta }_{M}\right]$ , $0 \le {\theta }_{m} \le 2\pi$ and $m = 1, 2, 3, . . . ,M$.

The synthesized array factor can be formulated in matrix notation to enable structured computation. Let ${\left[{\mathbf{W}}_{s}\right]}_{1\times N}$ denote the synthesized excitation coefficient vector, ${\left[{\mathbf{A}}_{s}\right]}_{N\times M}$ represent the synthesized steering matrix constructed across $M$ angular samples, and ${\left[{AF}_{s}\left(\theta \right)\right]}_{1\times M}$ be the synthesized array factor evaluated at those sample angles. These quantities are explicitly defined as:

17 $${\left[{\mathbf{W}}_{s}\right]}_{1\times N}=\left[{\widehat{w}}_{1},{\widehat{w}}_{2},{\widehat{w}}_{3},\dots ,{\widehat{w}}_{N}\right]$$

18 $${\left[{\mathbf{A}}_{s}\right]}_{N\times M}=\left[\begin{array}{ccc}1& 1 \cdots & 1\\ \begin{array}{c}{e}^{jk{d}_{s}\mathrm{cos}{\theta }_{1}}\\ . ..\end{array}& \begin{array}{c}{e}^{jk{d}_{s}\mathrm{cos}{\theta }_{2}}\\ . ..\end{array} \begin{array}{c} . ..\\ . ..\end{array}& \begin{array}{c}{e}^{jk{d}_{s}\mathrm{cos}{\theta }_{M}}\\ . ..\end{array}\\ {e}^{jk\left(N-1\right){d}_{s}\mathrm{cos}{\theta }_{1}}& {e}^{jk\left(N-1\right){d}_{s}\mathrm{cos}{\theta }_{2}} \cdots & {e}^{jk\left(N-1\right){d}_{s}\mathrm{cos}{\theta }_{M}}\end{array}\right]$$

19 $${\left[{AF}_{s}\left(\theta \right)\right]}_{1\times M}=\left[{AF}_{s}\left({\theta }_{1}\right) {AF}_{s}\left({\theta }_{2}\right){ AF}_{s}\left({\theta }_{3}\right)\dots {AF}_{s}\left({\theta }_{M}\right)\right]$$

Accordingly, the synthesized array factor can be written in matrix form as follows:

20 $$\left[ {AF_{s} \left( \theta \right)} \right]_{1 \times M} = \left[ {w_{s} } \right]_{1 \times N} \left[ {{\mathbf{A}}_{s} } \right]_{N \times M}$$

Using (16) and (20), the expression represented in (15) can be rewritten as follows:

21 $${{\left[{\mathbf{W}}_{s}\right]}_{1\times N} \left[{\mathbf{A}}_{s}\right]}_{N\times M}\approx {\left[{AF}_{d}\left(\theta \right)\right]}_{1\times M}$$

The vector ${\left[{AF}_{d}\left(\theta \right)\right]}_{1\times M}$, representing the desired array factor sampled over $M$ angular directions, is entirely known and analytically defined based on the raised cosine function. In contrast, the synthesized steering matrix ${\left[{A}_{s}\right]}_{N\times M}$ depends on the inter-element spacing ${d}_{s}$, which is not fixed a priori and must be optimized. Since ${\mathbf{A}}_{s}$ is a nonlinear function of the spacing configuration, determining its optimal structure becomes a part of the overall synthesis problem. Accordingly, the task of solving Eq. (21) is reduced to estimating the optimal excitation vector ${\left[{\mathbf{W}}_{s}\right]}_{1\times N}$. that best reproduces the desired pattern, given a spacing arrangement ${\mathbf{d}}_{s}$ optimized using the GA.

Multiply both sides of (21) by ${\left[{\mathbf{A}}_{s}\right]}_{\mathrm{M}\times N}^{H}$ which is defined as the Hermitian transpose of the matrix ${\left[{\mathbf{A}}_{s}\right]}_{N\times M}$ then

22 $$\left[ {{\mathbf{W}}_{s} } \right]_{1 \times N} \left[ {{\mathbf{A}}_{s} } \right]_{N \times M} \left[ {{\mathbf{A}}_{s} } \right]_{M \times N}^{H} = \left[ {AF_{d} \left( \theta \right)} \right]_{1 \times M} \left[ {{\mathbf{A}}_{s} } \right]_{{{\mathrm{M}} \times N}}^{H}$$

Let ${\left[\mathcal{R}\right]}_{N\times N}={\left[{\mathbf{A}}_{s}\right]}_{N\times M}{\left[{\mathbf{A}}_{s}\right]}_{\mathrm{M}\times N}^{H}$, then (23) is rewritten as:

23 $${\left[{\mathbf{W}}_{s}\right]}_{1\times N}{\left[\mathcal{R}\right]}_{N\times N}={\left[{AF}_{d}\left(\theta \right)\right]}_{1\times M}{\left[{\mathbf{A}}_{s}\right]}_{M\times N}^{H}$$

The synthesized excitation vector ${\left[{\mathbf{W}}_{s}\right]}_{1\times N}$ can be determined as follows:

24 $${\left[{\mathbf{W}}_{s}\right]}_{1\times N}= {\left[{AF}_{d}\left(\theta \right)\right]}_{1\times M}{\left[{\mathbf{A}}_{s}\right]}_{M\times N}^{H}{\left[\mathcal{R}\right]}_{N\times N}^{-1}$$

where ${\left[\mathcal{R}\right]}_{N\times N}^{-1}$ is the inverse of the matrix ${\left[\mathcal{R}\right]}_{N\times N}$.

The synthesized excitation vector ${\left[{\mathbf{W}}_{s}\right]}_{1\times N}$ can be obtained by solving the linear system defined in Eq. (24), which provides a closed-form solution for the optimal excitation coefficients corresponding to a given steering matrix configuration. In parallel, the GA is employed to determine the optimal values of the inter-element spacing vector ${\mathbf{d}}_{s}$, such that the synthesized array pattern achieves the lowest possible SLL. This dual-stage approach combining deterministic excitation synthesis with evolutionary spacing optimization ensures that the array simultaneously satisfies beam shaping accuracy and physical design constraints. Equation (10) defines the desired radiation pattern, while Eq. (24) provides the closed-form solution for the excitation coefficients that best synthesize this pattern for a given array geometry. To further enhance beam shaping flexibility and reduce sidelobe levels, the inter-element spacing vector $\mathbf{d}=[{d}_{1},{d}_{2},\dots ,{d}_{N-1}]$ is optimized using GA. The objective is to minimize composite cost function^29^:

25 $$\mathcal{C}(\mathbf{d})={\delta }_{1}\cdot {\mathrm{SLL}}_{\mathrm{max}}(\mathbf{d})+{\delta }_{2}\cdot \mathrm{HPBW}(\mathbf{d})+{\delta }_{3}\cdot \mathrm{Penalty}(\mathbf{d})$$

where $\mathcal{C}(\mathbf{d})$ denotes the total scalar cost to be minimized, ${\mathrm{SLL}}_{\mathrm{max}}(\mathbf{d})$ is the maximum sidelobe level (in dB), $\mathrm{HPBW}(\mathbf{d})$ is the half-power beamwidth of the synthesized main lobe, and $\mathrm{Penalty}(\mathbf{d})$ is a constraint function that penalizes violations of spacing limits. The weights ${\delta }_{1},{\delta }_{2},{\delta }_{3}$ are used to control the trade-off between objectives and are typically selected from the range [0.1, 1], normalized such that ${\delta }_{1}+{\delta }_{2}+{\delta }_{3}=1$.

The penalty term is defined to impose strict compliance with element spacing bounds and is formulated as:

26 $$\mathrm{Penalty}(\mathbf{d})=\sum_{i=1}^{N-1}\left[\xi \cdot \mathrm{max}(0,{d}_{i}-{d}_{\mathrm{max}}{)}^{2}+\eta \cdot \mathrm{max}(0,{d}_{\mathrm{min}}-{d}_{i}{)}^{2}\right]$$

Here, ${d}_{\mathrm{max}}$ and ${d}_{\mathrm{min}}$ represent the maximum and minimum allowable inter-element spacings, typically set within the range $0.25\lambda \le {d}_{i}\le \lambda$. The penalty weights $\xi$ and $\eta$ (usually between ${10}^{2}$ and ${10}^{4}$) modulate the severity of constraint violations, allowing fine control over optimization sensitivity.

Within the GA framework, each chromosome encodes a candidate spacing configuration $\mathbf{d}=[{d}_{1},{d}_{2},\dots ,{d}_{N-1}]$. For each candidate, the corresponding array factor is computed as:

27 $$AF\left( \theta \right) = \mathop \sum \limits_{n = 1}^{N} w_{n} e^{{jkx_{n} {\mathrm{cos}}\left( \theta \right)}} \;{\mathrm{with}}\;x_{n} = \mathop \sum \limits_{i = 1}^{n - 1} d_{i}$$

From this synthesized pattern, the values of ${\mathrm{SLL}}_{\mathrm{max}}$ and $\mathrm{HPBW}$ are extracted and used to evaluate the overall cost $\mathcal{C}(\mathbf{d})$. This cost guides the evolutionary selection process within the GA, steering the population toward configurations that achieve enhanced sidelobe suppression while satisfying practical spacing constraints and physical realizability.

Equation (10) defines the analytically constructed target pattern $A{F}_{RC}(\theta )$, derived from the raised-cosine spatial mapping. While this expression characterizes the ideal angular response, it does not yet include the excitation coefficients or physical array geometry required for practical implementation. Therefore, a clear synthesis procedure is necessary to transform this analytical pattern into a realizable antenna configuration.

First, the continuous raised-cosine-based pattern $A{F}_{d}(\theta )$, obtained directly from Eq. (10), is sampled at $M$ discrete angular points to form the desired response vector expressed in Eq. (16). which represents the numerical target to be synthesized. The array response is then modeled using the matrix relation in Eq. (20). Where $[{A}_{s}]$ is the steering matrix corresponding to the assumed array geometry defined by the spacing vector $\mathbf{d}=[{d}_{1},{d}_{2},\dots ,{d}_{N-1}]$. For any given spacing configuration, the steering matrix becomes known, enabling the synthesis problem to be solved as an inverse problem. The optimal excitation vector is obtained using the closed-form expression in Eq. (24), which provides the excitations that best approximate the desired RC-based pattern for that particular geometry.

Since the inter-element spacings directly affect the structure of $[{A}_{s}]$ and thus the synthesized pattern, the spacing vector $\mathbf{d}$ is treated as the main optimization variable. GA is employed to search the spacing domain. For each candidate spacing vector generated by the GA, the optimal excitations ${W}_{s}$ are computed analytically through the above matrix inversion, and the resulting pattern is evaluated using the cost function of Eq. (25), which balances sidelobe suppression, beamwidth control, and physical spacing constraints.

The overall synthesis procedure therefore consists of two integrated stages. The first stage defines the desired array factor analytically using the raised-cosine formulation of Eq. (10). The second stage realizes this pattern through a hybrid process in which the array geometry is optimized stochastically using the GA, while the corresponding excitation coefficients are computed deterministically via the matrix solution in Eq. (24). This hybrid mechanism establishes the complete link between the analytically constructed RC-based target function and the physically realizable array pattern.

Accordingly, the complete synthesis is performed through a two-stage hybrid strategy:

Analytical Pattern Construction The desired array factor $A{F}_{d}(\theta )$ is derived in closed form using the raised-cosine spatial mapping of Eq. (10).

The optimization process is implemented using a genetic algorithm to determine the optimal inter-element spacing. The main GA parameters used in this study are summarized as follows: the population size is set to 50 individuals, the maximum number of generations is 200, the crossover probability is 0.85, and the mutation rate is 0.05. Tournament selection is adopted to choose parent individuals, while an elitism strategy is applied to preserve the best solution in each generation. These parameters were selected to ensure stable convergence and effective exploration of the search space.

1. *Hybrid Synthesis of Excitations and Geometry*
  The array geometry (inter-element spacings) is optimized using GA.
  For each geometry candidate, the optimal excitation vector is computed deterministically via Eq. (24).
  The resulting pattern is compared against the desired RC-based pattern.

The practical implementation of Eq. (24) requires the inversion of the $\mathcal{R}$ matrix, which is a function of the array geometry and sampling angles. It is acknowledged that this matrix can become ill-conditioned, particularly for arrays with large inter-element spacing or an poorly chosen set of sample angles ${{\boldsymbol{\theta}}}_{{\boldsymbol{m}}}$. An ill-conditioned $\mathcal{R}$ matrix can lead to significant errors in the computed excitation coefficients $[{\mathbf{W}}_{\mathbf{s}}]$, resulting in a synthesized pattern that deviates from the desired target $[\mathbf{A}{\mathbf{F}}_{\mathbf{d}}]$. To mitigate this, two strategies are employed:

1. Angular Sampling Density: The number of sample points $M$ is chosen to be significantly larger than the number of elements $N$  and are distributed to ensure the steering matrix $[{\mathrm{A}}_{\mathrm{s}}]$ is well-conditioned.
2. Regularization within the GA: The genetic algorithm’s cost function (Eq. 25) inherently acts as a regularizer. Candidate spacing vectors $\mathrm{d}$ that lead to an ill-conditioned $\mathcal{R}$ matrix and produce a poor pattern fit (high SLL, distorted beam) are automatically penalized and selected against during the evolutionary process. This ensures the final optimized design is not only performance-driven but also numerically robust.

### Novelty of the proposed synthesis framework

It should be emphasized that the novelty of the proposed method does not stem from the raised cosine function itself, which has been widely used in signal processing. Instead, the innovation lies in the hybrid synthesis framework that integrates analytical spatial pattern construction with optimization-based geometry design.

The proposed approach offers several advantages compared with conventional antenna array synthesis techniques:

1. Deterministic radiation pattern construction using analytical spatial shaping.
2. Closed-form computation of excitation coefficients, avoiding iterative optimization.
3. Reduced optimization dimensionality, since only the array geometry is optimized.
4. Improved computational efficiency compared with fully stochastic synthesis methods.
5. Enhanced control of the SLL–beamwidth trade-off through the roll-off parameter.

These features distinguish the proposed method from traditional window-based amplitude tapering and purely optimization-based array synthesis techniques.

## Simulation results of proposed technique

**Fig. 2:**
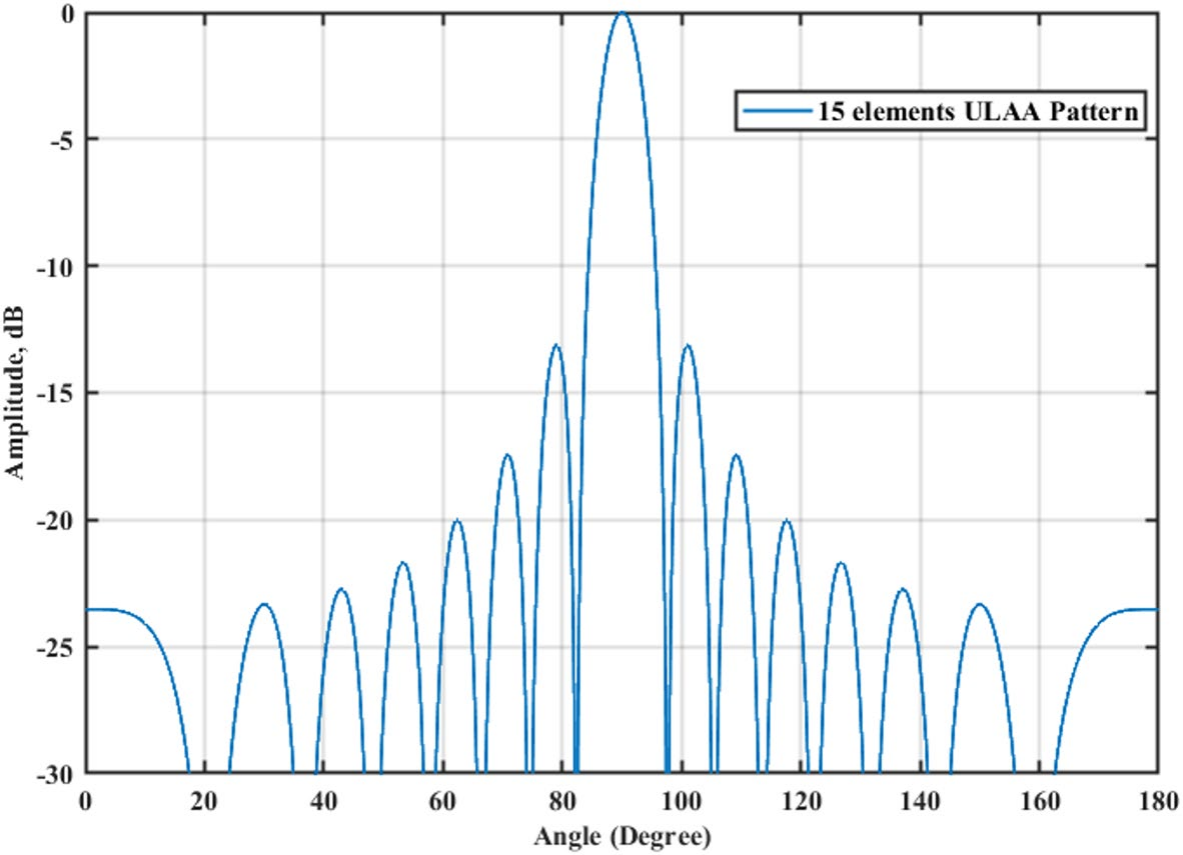
MATLAB-generated 2D radiation pattern of the $15$-element broadside ULAA.

**Fig. 3:**
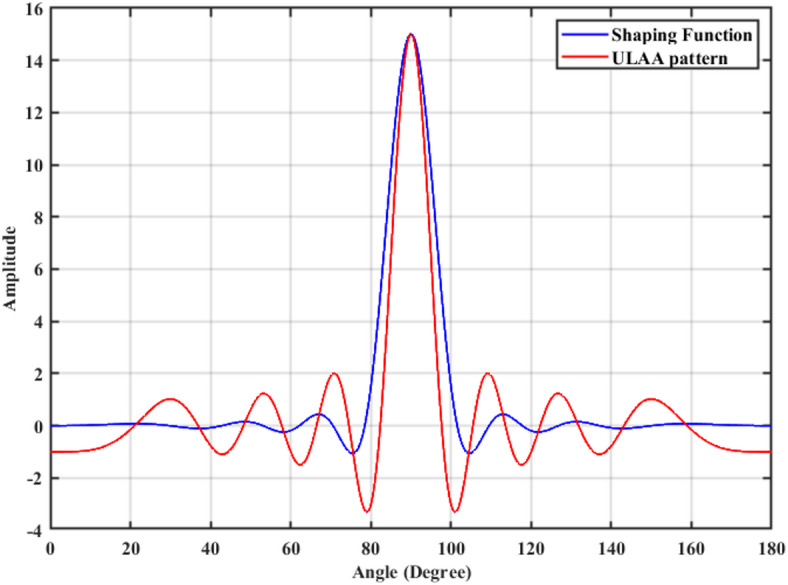
Radiation pattern of the $N=15$ elements ULAA alongside the applied shaping function.

**Fig. 4:**
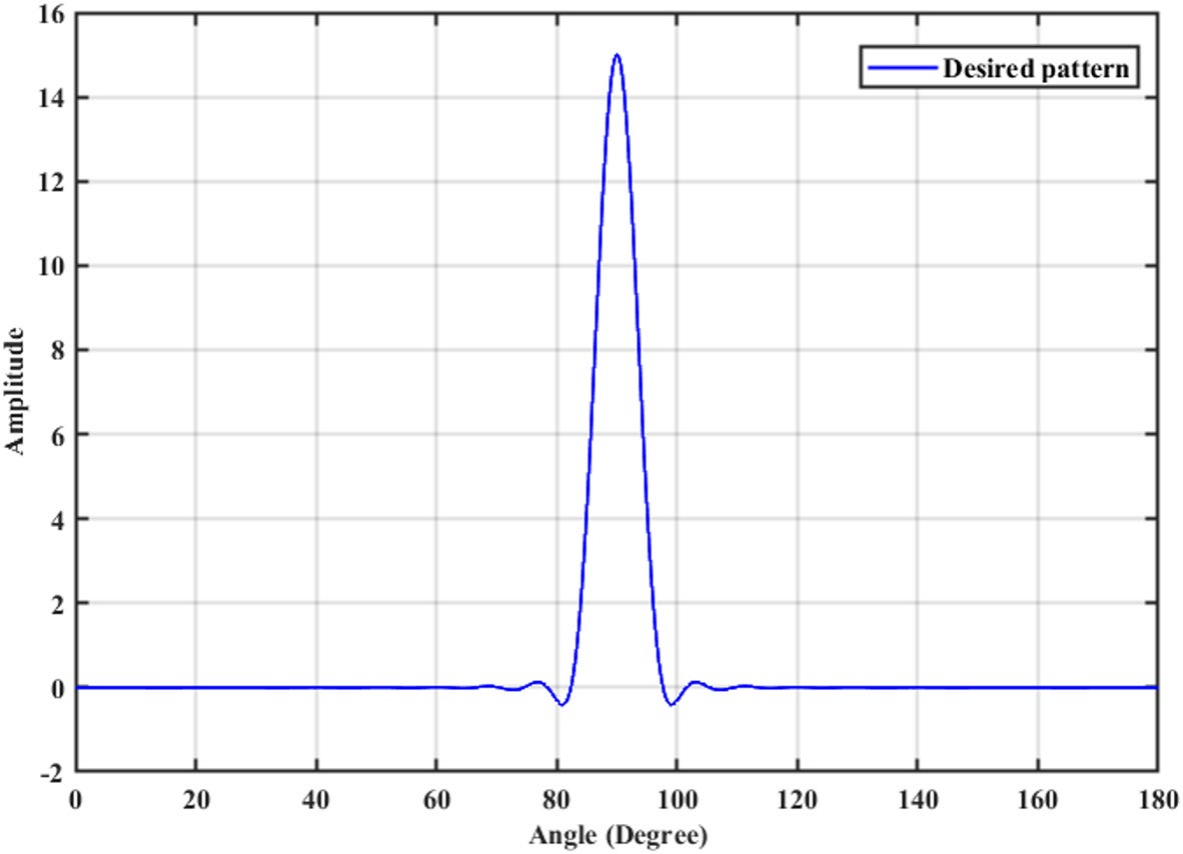
Desired radiation pattern synthesized for $N=15$ elements with roll-off $\beta =1$.

**Fig. 5:**
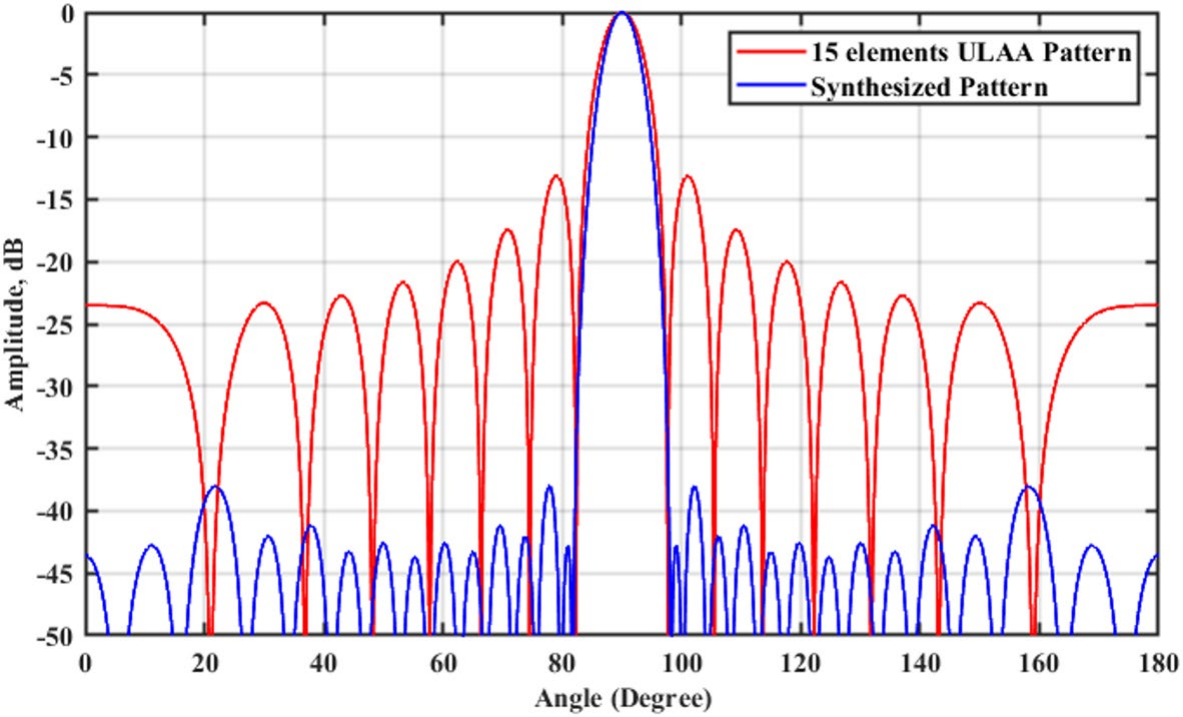
Radiation pattern of the synthesized array using the RCS-GA technique for $\beta =1$, compared to the ULAA pattern.

A 15-element broadside ULAA with half-wavelength spacing $d_{x} = \lambda /2$ is initially evaluated to establish reference performance. As shown in Fig. 2, the two-dimensional radiation pattern of the conventional ULAA exhibits a SLL of − 13.131 dB and a HPBW of $6.786^{^\circ } ,$ indicating moderate directivity and limited interference suppression capability. To enhance radiation performance, a shaping function derived from the RC profile is applied to modulate the array factor of the ULAA, as formulated in Eq. (15). This shaping function—analytically extracted from the RC envelope—acts as a spatial taper that suppresses sidelobes while preserving the main-lobe structure. Using a GA implemented in MATLAB, the optimal uniform inter-element spacing is determined to be $d_{s} = 0.83\lambda$. The resulting shaped radiation pattern, synthesized using the RC-modulated excitation profile, is shown in Fig. 3 alongside the applied shaping function. The analytically generated target pattern is presented in Fig. 4 for comparison. The final synthesized array, depicted in Fig. 5, achieves significant suppression of sidelobe energy while maintaining a narrow main lobe. Specifically, a First sidelobe level (FSLL) of $- 42.8\;{\mathrm{dB}}$ is attained, highlighting its effectiveness in mitigating interference and improving directivity.

A comprehensive comparison of the synthesized and conventional ULAA patterns is provided in Tables 1 and 2, examining critical parameters such as excitation coefficients, element spacing, dynamic range ratio (DRR), SLL, FSLL, HPBW, and null to null beamwidth (NNBW) for different values of the shaping parameter ($\beta = 1, 2,\;{\mathrm{and}}\;3$). The results indicate that the synthesized arrays achieve significant SLL suppression, reducing it to $- 38.05\;{\mathrm{dB}}$—nearly three times lower than the original ULAA’s SLL of $- 13.131\;{\mathrm{dB}}$—while maintaining a consistent HPBW of $5.526^\circ$. These findings underscore the potential of the proposed approach for applications requiring high-fidelity beam shaping and enhanced interference suppression.

**Table 1:** Comparative analysis of element spacing, synthesized $HPBW_{{\mathrm{s}}}$, $SLL_{{\mathrm{s}}}$, NNBW_s_, $FSLL_{{\mathrm{s}}}$, and $DRR_{{\mathrm{s}}}$ for the synthesized array versus the $15$-element ULAA.

15-element ULAA		Synthesized array using the RCS-GA algorithm $\beta =1$	
$N=15$ elements		*N* = 15 elements	
$d=0.878\lambda$		${d}_{s}=0.5\lambda$	
${w}_{1}$	1	${\widehat{w}}_{1}$	0.096
${w}_{2}$	1	${\widehat{w}}_{2}$	0.197
${w}_{3}$	1	${\widehat{w}}_{3}$	0.34
${w}_{4}$	1	${\widehat{w}}_{4}$	0.51
${w}_{5}$	1	${\widehat{w}}_{5}$	0.687
${w}_{6}$	1	${\widehat{w}}_{6}$	0.844
${w}_{7}$	1	${\widehat{w}}_{7}$	0.955
${w}_{8}$	1	${\widehat{w}}_{8}$	1
${w}_{9}$	1	${\widehat{w}}_{9}$	0.955
${w}_{10}$	1	${\widehat{w}}_{10}$	0.844
${w}_{11}$	1	${\widehat{w}}_{11}$	0.687
${w}_{12}$	1	${\widehat{w}}_{12}$	0.51
${w}_{13}$	1	${\widehat{w}}_{13}$	0.34
${w}_{14}$	1	${\widehat{w}}_{14}$	0.197
${w}_{15}$	1	${\widehat{w}}_{15}$	0.096
$HPBW_{s} = 5.526^{^\circ }$		${\mathrm{HPBW}} = 6.66^{^\circ }$	
$SLL_{s} = - 38.05\;{\mathrm{dB}}$		$FSLL_{s} = - 42.8\;{\mathrm{dB}}$ $SLL = - 13.131\;{\mathrm{dB}}$	
$NNBW_{s} = 16.524^{^\circ }$		$NNBW = 15.336^{^\circ }$	
$DRR_{s} = 10.42$		$DRR = 1$

**Table 2:** Comparative analysis of element spacing, synthesized $HPBW_{{\mathrm{s}}}$, $SLL_{{\mathrm{s}}}$, NNBW_s_, $FSLL_{{\mathrm{s}}}$, and $DRR_{{\mathrm{s}}}$ for the synthesized arrays with $\beta = 2\;{\mathrm{and}}\;\beta = 3$.

Synthesized array using the RCS-GA algorithm $\beta =3$		Synthesized array using the RCS-GA algorithm $\beta =2$	
$N=15$ elements		$N=15$ elements	
${d}_{s}=0.9\lambda$		${d}_{s}=0.899\lambda$	
${\widehat{w}}_{1}$	0.3287	${\widehat{w}}_{1}$	0.2432
${\widehat{w}}_{2}$	0.3967	${\widehat{w}}_{2}$	0.3381
${\widehat{w}}_{3}$	0.5133	${\widehat{w}}_{3}$	0.4708
${\widehat{w}}_{4}$	0.6486	${\widehat{w}}_{4}$	0.6189
${\widehat{w}}_{5}$	0.7823	${\widehat{w}}_{5}$	0.7634
${\widehat{w}}_{6}$	0.8955	${\widehat{w}}_{6}$	0.8857
${\widehat{w}}_{7}$	0.9719	${\widehat{w}}_{7}$	0.9688
${\widehat{w}}_{8}$	1	${\widehat{w}}_{8}$	1
${\widehat{w}}_{9}$	0.9719	${\widehat{w}}_{9}$	0.9688
${\widehat{w}}_{10}$	0.8955	${\widehat{w}}_{10}$	0.8857
${\widehat{w}}_{11}$	0.7823	${\widehat{w}}_{11}$	0.7634
${\widehat{w}}_{12}$	0.6486	${\widehat{w}}_{12}$	0.6189
${\widehat{w}}_{13}$	0.5133	${\widehat{w}}_{13}$	0.4708
${\widehat{w}}_{14}$	0.3967	${\widehat{w}}_{14}$	0.3381
${\widehat{w}}_{15}$	0.3287	${\widehat{w}}_{15}$	0.2432
$HPBW_{s} = 4.806^{^\circ }$		$HPBW_{s} = 4.59^{^\circ }$	
$SLL_{s} = - 29.05\;{\mathrm{dB}}$		$SLL_{s} = - 26.1\;{\mathrm{dB}}$	
$FSLL_{s} = - 30.16\;{\mathrm{dB}}$		$FSLL_{s} = - 27.18\;{\mathrm{dB}}$	
$NNBW_{s} = 12.528^{^\circ }$		$NNBW_{s} = 11.88^{^\circ }$	
$DRR_{s} = 4.11$		$DRR_{s} = 3.04$	

**Fig. 6:**
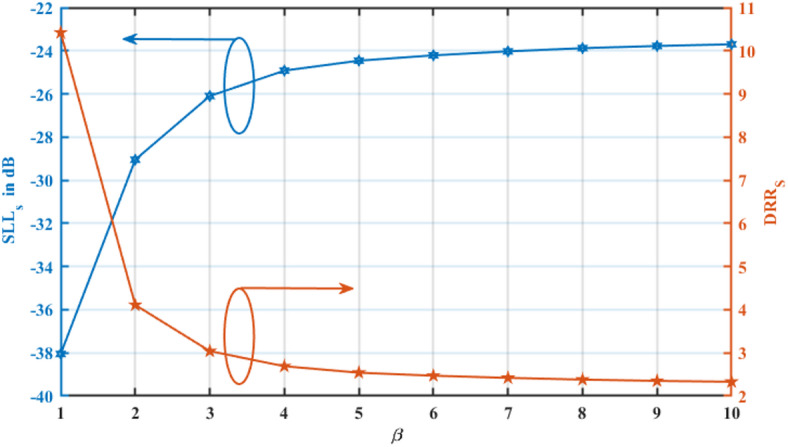
Influence of the β parameter on $SLL_{{\mathrm{s}}}$ and $DRR_{{\mathrm{s}}}$.

The analysis presented in Fig. 6 provides a comprehensive evaluation of the influence of the β parameter on the synthesized sidelobe level $(SLL_{{\mathrm{s}}}$) and synthesized dynamic range ratio ($DRR_{{\mathrm{s}}}$) in the antenna array. The blue curve represents the variation of $SLL_{{\mathrm{s}}}$ (in dB), while the red curve illustrates the corresponding DRR_s_ values. The observed trend reveals that as $\beta$ increases, SLL_s_ deteriorates, indicating a rise in sidelobe levels. In contrast, the DRR_s_ declines significantly with increasing β, demonstrating that higher β values yield a narrower main lobe, albeit at the cost of elevated sidelobe levels.

This fundamental trade-off plays a crucial role in practical antenna array applications. A smaller $\beta$ value enhances sidelobe suppression, making it highly beneficial for reducing interference in wireless communication and radar systems. On the other hand, a larger $\beta$ value improves directivity, which is particularly advantageous in applications requiring high-resolution imaging and long-range target detection.

**Fig. 7:**
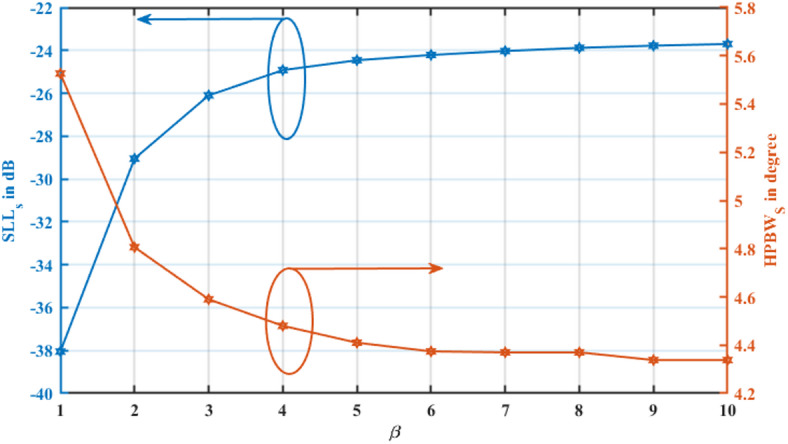
Influence of the β parameter on $SLL_{{\mathrm{s}}}$ and $HPBW_{{\mathrm{s}}}$.

The effects of β variation are further examined in Fig. 7, which illustrates its impact on both SLL and HPBW. The blue curve exhibits an increasing trend in SLL_s_, reinforcing the observation that higher β values lead to a degradation in sidelobe suppression. Meanwhile, the orange curve demonstrates a decreasing trend in HPBW, confirming that higher $\beta$ values enhance beam sharpness and directivity.

The findings presented in Figs. 6 and 7 align with established principles of array synthesis, highlighting the intricate balance between beamwidth control and sidelobe suppression. This trade-off remains a critical consideration in modern phased array systems and adaptive beamforming technologies, where optimizing both parameters is essential for achieving superior performance in next-generation wireless, radar, and imaging applications.

### Comparative analysis of SLL reduction using MOM/GA and RCS-GA techniques

SLL suppression and HPBW reduction are critical objectives in linear antenna array design, particularly for applications requiring high directivity and minimal interference. This study presents a detailed comparison between rectangular window-based shaping functions and raised cosine window-based shaping functions, both implemented within an RCS-GA and GA-based optimization framework. The goal is to evaluate the impact of these shaping functions on radiation pattern control, element spacing, and excitation coefficients. The original 15-element ULAA, before optimization, exhibits a SLL of $- 13.131\;{\mathrm{dB}}$ and a HPBW of $6.66^\circ$. These values indicate that while the ULAA provides acceptable radiation performance, it suffers from high sidelobe levels, which can lead to interference and reduced beam efficiency.

The first optimization approach applies the rectangular window shaping function within the MoM/GA framework as introduced in^30^. As shown in the simulation results in Table 3, the optimized element spacing values are obtained, and the radiation characteristics improve significantly. The SLL is reduced to $-52.84$ dB, indicating a substantial improvement over the original ULAA. The HPBW is also reduced to 5.76°, showing an increase in beam directivity. However, the limitation of this approach is that the FSLL remains relatively high, and the main beam experiences minor distortions, leading to an imbalance between sidelobe suppression and beam shaping. In contrast, the raised cosine window-based RCS-GA technique provides a more structured and effective shaped approach. By adjusting the shaping function parameters and optimizing the element spacing and excitation coefficients, the SLL is significantly reduced to $-38.05$ dB, which is a substantial improvement over both the original ULAA and the rectangular window approach. Furthermore, the HPBW is optimized to $5.526^\circ$, offering sharper beam focusing with lower sidelobes. The FSLL is also suppressed to $-42.8$ dB, demonstrating the superior sidelobe attenuation capabilities of the raised cosine shaping function.

**Table 3:** Comparative analysis of element spacing, synthesized $HPBW_{{\mathrm{s}}}$, $SLL_{{\mathrm{s}}}$, NNBW_s_, $FSLL_{{\mathrm{s}}}$, and $DRR_{{\mathrm{s}}}$ for the synthesized rectangular window-based MoM/GA with ULAA.

15 elements ULAA		Synthesized array using the MoM/GA algorithm rectangular window	
$N=15$ elements		$N=15$ elements	
$d=0.5\lambda$		${d}_{s}=0.8\lambda$	
${w}_{1}$	1	${\widehat{w}}_{1}$	0.2364
${w}_{2}$	1	${\widehat{w}}_{2}$	0.6783
${w}_{3}$	1	${\widehat{w}}_{3}$	1.4616
${w}_{4}$	1	${\widehat{w}}_{4}$	2.5793
${w}_{5}$	1	${\widehat{w}}_{5}$	3.897
${w}_{6}$	1	${\widehat{w}}_{6}$	5.164
${w}_{7}$	1	${\widehat{w}}_{7}$	6.08
${w}_{8}$	1	${\widehat{w}}_{8}$	6.42
${w}_{9}$	1	${\widehat{w}}_{9}$	6.08
${w}_{10}$	1	${\widehat{w}}_{10}$	5.164
${w}_{11}$	1	${\widehat{w}}_{11}$	3.897
${w}_{12}$	1	${\widehat{w}}_{12}$	2.5793
${w}_{13}$	1	${\widehat{w}}_{13}$	1.4616
${w}_{14}$	1	${\widehat{w}}_{14}$	0.6783
${w}_{15}$	1	${\widehat{w}}_{15}$	0.2364
${\mathrm{HPBW}} = 6.66^{^\circ }$		$HPBW_{s} = 6.66^{^\circ }$	
$SLL = - 13.131\;{\mathrm{dB}}$		$SLL_{s} = - 53.11\;{\mathrm{dB}}$ $FSLL_{s} = - 55.54\;{\mathrm{dB}}$	
$NNBW = 15.336^{^\circ }$		$NNBW_{s} = 24.84$	
$DRR = 1$		$DRR_{s} = 27.17$	

The effect of the $\beta$ parameter is analyzed to determine its influence on SLL suppression, HPBW reduction, and directivity control. The key observations from the numerical results are:

- For $\beta = 1$, the synthesized array achieves SLL = -38.05 dB, FSLL = -42.8 dB, and HPBW = 5.526°, providing an optimal balance between sidelobe suppression and beam sharpness.
- For $\beta = 2$, the SLL degrades to -29.05 dB, with an FSLL of -30.16 dB, while the HPBW is further reduced to 4.806°, demonstrating a trade-off between sidelobe reduction and beam narrowing.
- For $\beta = 3$, SLL increases further to -26.1 dB, with HPBW reaching 4.126°, emphasizing a preference for beam directivity at the expense of higher sidelobe levels.

The comparative analysis between the rectangular window and raised cosine window approaches is summarized in the numerical results tables, where key parameters such as element spacing, HPBW, SLL, FSLL, and DRR are evaluated. The findings confirm that:

- The rectangular window-based approach achieves moderate sidelobe suppression, but its HPBW remains relatively broad, making it less suitable for applications requiring sharp beam control.
- The raised cosine window-based approach significantly improves SLL reduction, achieving a well-balanced trade-off between beam sharpness and sidelobe attenuation, making it the preferred method for high-precision radar, electronic warfare, and microwave imaging applications.

The synthesized radiation pattern using the GA achieves a SLL of $- 53.1121\;{\mathrm{dB}}$ and a HPBW of $6.66^\circ$. The optimization parameters include element spacing $d_{s} = 0.8 \lambda$, exponent $n = 1.3$, and additional height $h = 4.2$. The shaping function parameters are set with a main beam direction of $\phi_{o} = 90^{^\circ }$ and a beamwidth control factor of $w = 23^{^\circ }$. parameters are adjusted such that $\phi_{o} = 90^{^\circ }$, and $w = 23^{^\circ }$.

The comparative analysis presented in Table 4 highlights the performance trade-offs between the proposed RCS-GA algorithm and the conventional MoM/GA algorithm with a rectangular window. The RCS-GA algorithm, evaluated across three parameter settings ($\beta =1,2,3$), demonstrates distinct advantages in beamwidth control but exhibits limitations in side-lobe suppression and dynamic range adaptability. Specifically, the RCS-GA achieves a narrower HPBW_s_ ranging from ${4.59}^{\circ }$ to ${5.56}^{\circ }$, compared to ${6.66}^{\circ }$ for the MoM/GA, indicating superior directivity. However, this improvement comes at the cost of elevated SLL_s_ and FSLL_s_, which are critical for interference mitigation. For instance, the SLL_s_ for RCS-GA ranges between $-26.1$ dB and $-38.05$ dB, significantly higher than the $-53.11$ dB achieved by MoM/GA. This suppression is crucial for minimizing long-range interference, clutter returns, and spurious detections in wide-field radar or imaging systems. The FSLL_s_ are also effectively reduced by the RCS-GA approach, reaching as low as $-42.8$ dB. Nonetheless, extremely low FSLL_s_ imposes tighter constraints on the system’s dynamic range ratio and amplifier linearity. Accurate detection of weak far-field signals in the presence of strong main beam energy demands a highly linear and high-resolution RF front-end, potentially increasing system complexity and cost. Similarly, the NNBW_s_ is narrower for RCS-GA (${11.8}^{\circ }$ to ${16.52}^{\circ }$ versus ${24.84}^{\circ }$), suggesting better angular resolution but potential challenges in null steering applications. Such narrower null spacing improves angular discrimination and provides enhanced interference isolation, particularly in applications involving multiple adjacent sources or jamming scenarios. However, this advantage introduces trade-offs. Tightly spaced nulls become more sensitive to mechanical tolerances, phase inconsistencies, and fabrication errors, potentially compromising the robustness of null steering in adaptive beamforming or real-time direction-of-arrival (DOA) systems. Furthermore, the DRR_s_ of RCS-GA ($3.04$ to $10.42$) is markedly lower than that of MoM/GA ($27.17$), limiting its effectiveness in environments with high signal variability. A low DRR_s_ signifies strong main lobe concentration and excellent suppression of undesired emissions, which is vital for applications requiring clean spectral footprints and narrow detection fields. However, achieving and maintaining high DRR_s_ in hardware requires advanced digital-to-analog converters (DACs), high-fidelity power amplifiers, and precise calibration, which can significantly increase power consumption and implementation cost. These results underscore a fundamental trade-off: while RCS-GA enhances beam focusing, it compromises on interference suppression and dynamic range, which are crucial for many practical applications. Future research should explore hybrid optimization techniques to balance these competing objectives, potentially leveraging the strengths of both algorithms to achieve a more robust solution.

**Table 4:** Comparative analysis of element spacing, synthesized $HPBW_{{\mathrm{s}}}$, $SLL_{{\mathrm{s}}}$, NNBW_s_, $FSLL_{{\mathrm{s}}}$, and $DRR_{{\mathrm{s}}}$ for the synthesized rectangular window-based MoM/GA versus the proposed RCS-GA algorithm.

Synthesized array using the MoM/GA algorithm rectangular window	Synthesized array using the RCS-GA algorithm $\beta =1$	Synthesized array using the RCS-GA algorithm $\beta =2$	Synthesized array using the RCS-GA algorithm $\beta =3$
$N=15$ elements	$N=15$ elements	$N=15$ elements	$N=15$ elements
$d_{s} = 0.8\lambda$	$d_{s} = 0.878\lambda$	$d_{s} = 0.899\lambda$	$d_{s} = 0.9\lambda$
$HPBW_{s} = 6.66^{^\circ }$	$HPBW_{s} = 5.526^{^\circ }$	$HPBW_{s} = 4.806^{^\circ }$	$HPBW_{s} = 4.59^{^\circ }$
$SLL_{s} = - 53.11\;{\mathrm{dB}}$	$SLL_{s} = - 38.05\;{\mathrm{dB}}$	$SLL_{s} = - 29.05\;{\mathrm{dB}}$	$SLL_{s} = - 26.1\;{\mathrm{dB}}$
$FSLL_{s} = - 55.54\;{\mathrm{dB}}$	$FSLL_{s} = - 42.8\;{\mathrm{dB}}$	$FSLL_{s} = - 30.16\;{\mathrm{dB}}$	$FSLL_{s} = - 27.18\;{\mathrm{dB}}$
$NNBW_{s} = 24.84^{^\circ }$	$NNBW_{s} = 16.524^{^\circ }$	$NNBW_{s} = 12.528^{^\circ }$	$NNBW_{s} = 11.88^{^\circ }$
$DRR_{s} = 27.17$	$DRR_{s} = 10.42$	$DRR_{s} = 4.11$	$DRR_{s} = 3.04$

The DRR quantifies the ratio between the maximum and minimum excitation amplitudes required to realize a synthesized radiation pattern and directly impacts both hardware feasibility and pattern control capability. In the conventional MoM/GA-based uniform linear antenna array, a $DRR = 1$ is obtained due to uniform excitation, which represents a clear hardware advantage, as it minimizes amplifier dynamic range requirements and enhances tolerance to quantization errors and nonlinear effects. However, this extremely low DRR significantly limits the available amplitude shaping freedom, resulting in relatively poor sidelobe suppression, as evidenced by the achieved sidelobe level of only $-13.13$ dB.

In contrast, the proposed RCS-GA approach yields a higher but still practical $DRR_{{\mathrm{s}}} = 10.42$, reflecting the controlled non-uniform excitation required to achieve enhanced radiation performance. This moderate increase in DRR enables substantially improved sidelobe control, leading to a maximum synthesized sidelobe level of $-38.05$ dB and far sidelobe suppression down to $-42.8$ dB, while maintaining a narrow main lobe. Importantly, the obtained DRR_s_ remains within a realizable range for modern RF front-end hardware, preserving implementation feasibility. These results demonstrate that the proposed method achieves an effective compromise between hardware simplicity and radiation performance, where a modest increase in DRR yields a significant improvement in sidelobe suppression and beam shaping accuracy.

**Fig. 8:**
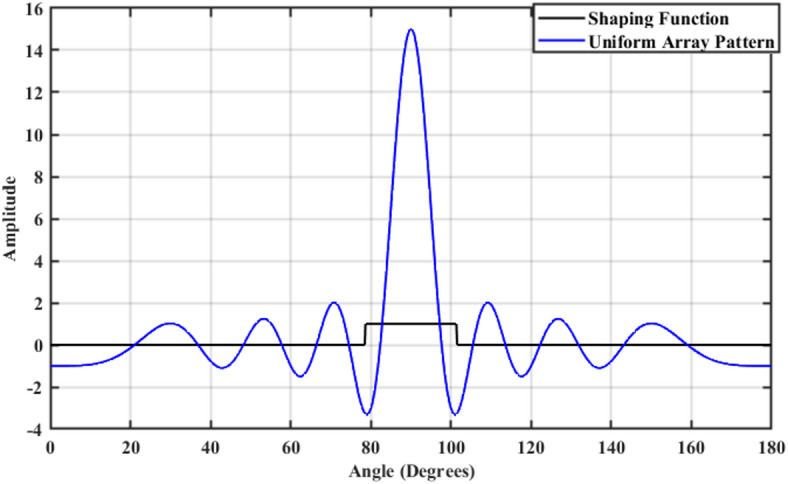
The 15-element uniform linear array pattern versus shaping function.

Figure 8 illustrates the radiation pattern of a 15-element ULAA (blue curve) and the applied shaping function (black curve). The uniform array pattern exhibits high sidelobe levels, which can lead to unwanted interference. The shaping function is employed to modify the array factor, ensuring SLL suppression while maintaining the integrity of the main beam. The effectiveness of the shaping function in constraining the sidelobe levels can be observed in controlled sidelobe behavior compared to the unshaped uniform array pattern.

**Fig. 9:**
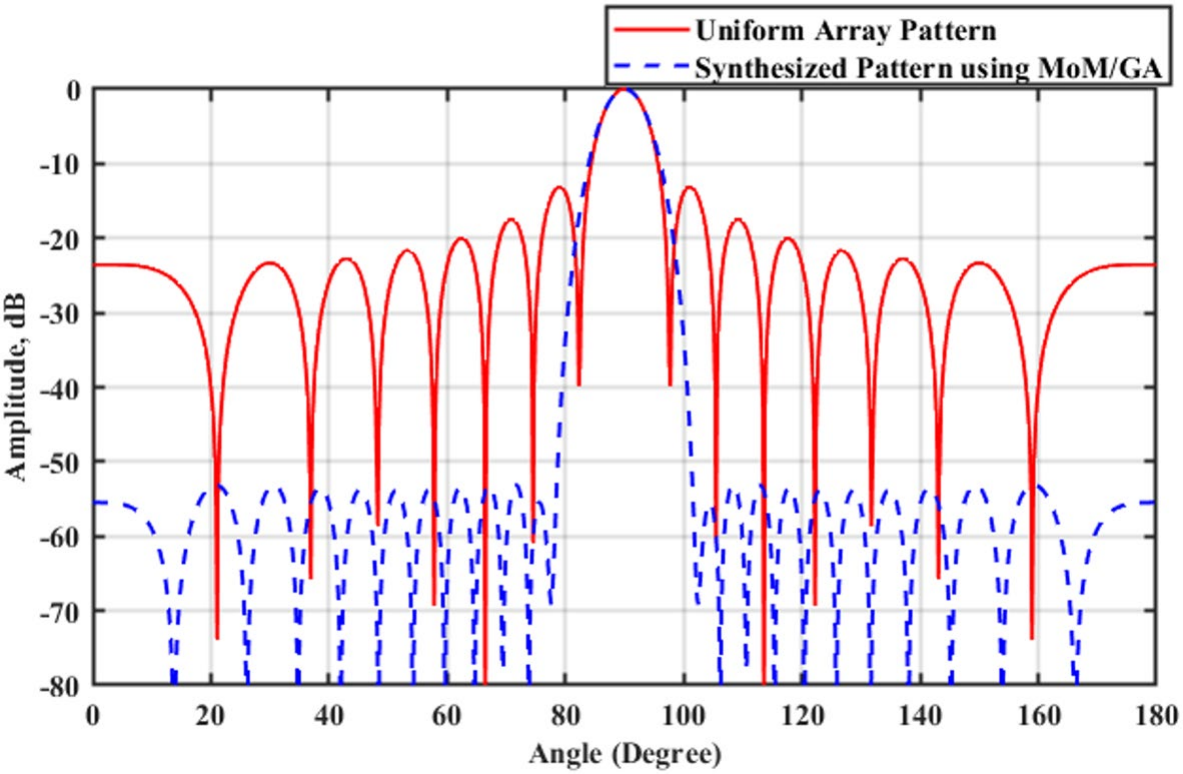
Comparison of radiation patterns in the 15-element ULAA versus MoM/GA Technique.

In Fig. 9, the red line solid represents the the uniform array pattern, while the blue dashed line shows the synthesized pattern using the MoM/GA. The synthesized pattern achieves lower sidelobes, enhancing beamforming performance.

**Fig. 10:**
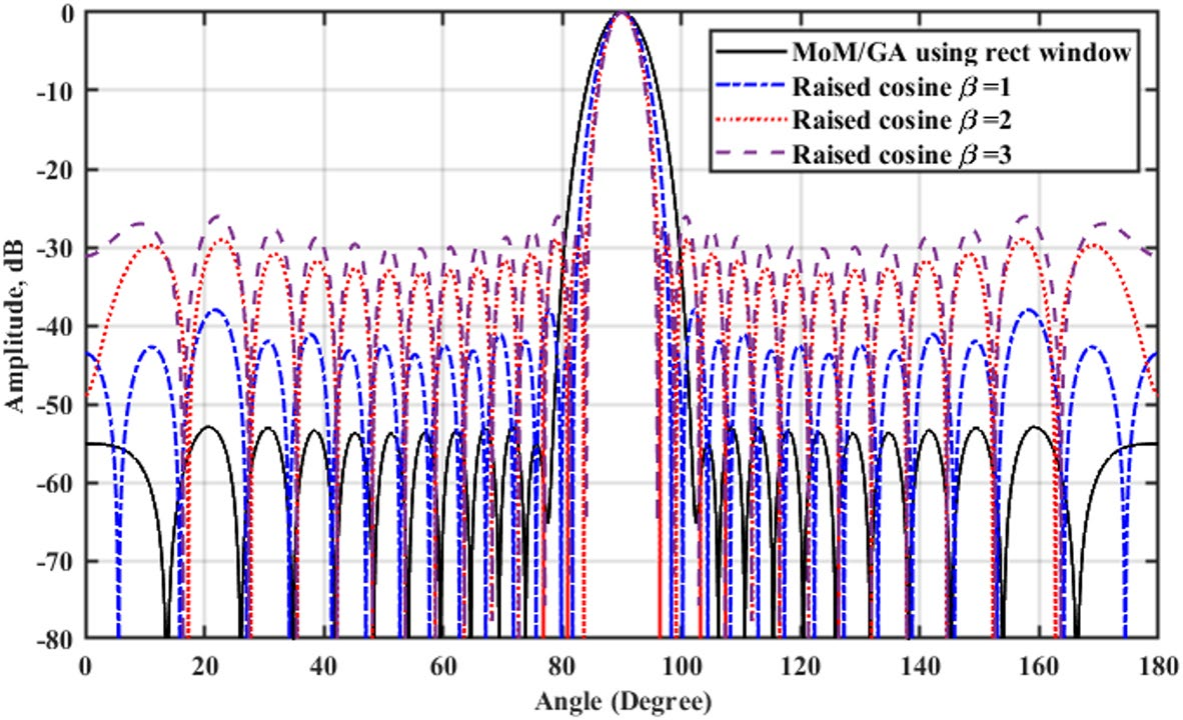
Comparison of radiation patterns the synthesized rectangular window based MoM/GA versus the proposed RCS-GA algorithm.

In Fig. 10. The black solid line represents the synthesized pattern using the MoM/GA with a rectangular window. The blue dashed, red dotted, and purple dashed lines correspond to raised cosine windows with $\beta =1$, $\beta =2$ and $\beta =3$, respectively.

An increase in the roll-off factor $\beta$ results in a narrower main lobe accompanied by higher sidelobe levels, thereby emphasizing the fundamental trade-off between beamwidth and sidelobe suppression.

### Elaboration on the SLL-beamwidth trade-off in RCS-GA

A fundamental challenge in antenna array synthesis is the inherent trade-off between sidelobe suppression and main beam narrowing. This trade-off is governed by the classical Fourier transform relationship between the array excitation distribution (aperture field) and the resulting far-field radiation pattern. In general, tapering the excitation amplitudes to reduce sidelobes broadens the main beam, while concentrating energy to narrow the beam inevitably raises sidelobe levels. The proposed EWASA framework addresses this trade-off through two complementary mechanisms: the analytically tunable roll-off factor $\beta$ and the hybrid GA-based spacing optimization.

#### The role of the roll-off factor ${\beta}$

The roll-off factor $\beta$ in the raised cosine shaping function (Eq. 11) serves as the primary control parameter for navigating the SLL-beamwidth trade-off. Mathematically, $\beta$ determines the excess bandwidth in the spatial frequency domain, which translates directly to the steepness of the main lobe transition and the rate of sidelobe decay.

- *For low* $\beta$ *values* ($\beta \to 0$): The shaping function approaches a sinc-like response, characterized by a relatively broader main lobe but significantly lower sidelobe levels. This regime prioritizes interference suppression and is optimal for applications requiring high signal-to-interference ratio, such as communication systems in dense spectral environments.
- *For high* $\beta$ *values* ($\beta \to 1$): The shaping function exhibits a more gradual roll-off, resulting in a narrower main lobe at the expense of elevated sidelobe levels. This regime prioritizes angular resolution and is advantageous for high-precision imaging and radar systems where target discrimination is paramount.

The results in Tables 1, 2, and Figs. 6–7 quantitatively illustrate this behavior. For $\beta =1$, the synthesized array achieves an SLL of $-38.0$ 5 dB with an HPBW of ${5.526}^{\circ }$, offering an excellent balance suitable for general-purpose applications. As $\beta$ increases to $2\;{\mathrm{and}}\;3$, the HPBW progressively narrows to ${4.806}^{\circ }$ and ${4.59}^{\circ }$, respectively, while the SLL rises to -29.05 dB and -26.1 dB. This monotonic relationship confirms that $\beta$ provides a continuous, predictable mechanism for tuning the array’s performance characteristics to meet specific system requirements.

#### The role of GA-based spacing optimization

While $\beta$ controls the *target* pattern characteristics, the genetic algorithm optimization of inter-element spacing provides an additional degree of freedom to partially mitigate the inherent trade-off. By allowing non-uniform spacing, the GA can achieve a more favorable SLL-beamwidth Pareto front than is possible with uniformly spaced arrays using amplitude tapering alone.

The cost function defined in Eq. (26) explicitly balances SLL and HPBW objectives through the weighting factors ${w}_{1}$ and ${w}_{2}$. This multi-objective optimization approach enables the algorithm to explore the design space and identify configurations that push the performance envelope beyond what is achievable with conventional windowing techniques. As demonstrated in Table 4, the RCS-GA algorithm achieves a superior combination of narrow beamwidth and low SLL compared to the rectangular window-based MoM/GA approach, which exhibits a broader main lobe despite its excellent sidelobe suppression.

#### Practical implications and application-specific tuning

The tunable nature of the EWASA framework allows designers to select the optimal operating point based on application-specific priorities:

- *Electronic Warfare and Radar*: For applications requiring high angular resolution to discriminate closely spaced targets, higher $\beta$ values (e.g., $\beta =2$ or $3$) may be preferred despite the moderate increase in sidelobe levels, provided that the system’s dynamic range and signal processing can tolerate the elevated interference.
- *Microwave Medical Imaging*: For applications where minimizing artifacts and false detections is paramount, lower $\beta$ values (e.g., $\beta =1$) that prioritize deep sidelobe suppression are more appropriate, even at the cost of slightly broader beamwidth.
- *Adaptive Beamforming Systems*: In scenarios with dynamic interference environments, the ability to adjust $\beta$ in software provides a flexible mechanism for reconfiguring the array’s radiation characteristics without physical modification.

#### Comparison with fundamental limits

It is instructive to compare the achieved SLL-beamwidth combinations with the theoretical limits imposed by the array aperture size. For a 15-element array with total length $L\approx (N-1)d$, the minimum achievable HPBW is approximately ${50.8}^{\circ }\lambda /L$ (in radians). For our optimized spacings (${d}_{s}\approx 0.878\lambda$), the total aperture length is approximately $12.3\lambda$, yielding a theoretical minimum HPBW of about ${4.13}^{\circ }$. The synthesized HPBW of ${5.526}^{\circ }$ for $\beta =1$ approaches this fundamental limit while simultaneously achieving excellent sidelobe suppression, demonstrating the efficiency of the proposed synthesis approach.

### Comparative analysis with classical tapering techniques

To rigorously evaluate the performance of the proposed RCS-GA, this subsection presents a detailed comparison with established amplitude tapering methods specifically designed for SLL control. All techniques are evaluated for a 15-element linear array with the same total aperture length to ensure fair comparison^31–33^ (Table 5).

**Table 5:** Performance comparison of RCS-GA with classical tapering techniques for 15-element linear arrays.

Tapering technique	SLL (dB)	HPBW (°)	FNBW (°)	DRR	Key characteristics
Uniform	$-13.2$	$6.79$	$15.34$	$1.0$	Baseline reference
Chebyshev (− 30 dB design)	$-30.0$	$6.12$	$14.28$	$\sim 5.2$	Equal sidelobes, narrowest beamwidth for given SLL
Taylor (${\overline{\mathrm{n}}}$ = 4, − 30 dB)	$-30.2$	$6.24$	$14.56$	$\sim 4.8$	Tapered sidelobes, better directivity
Binomial	$-30.6$	$8.45$	$18.92$	$\mathrm{High}$	Very smooth taper, broad beam
Cosine	$-23.5$	$7.12$	$16.34$	$3.2$	Moderate SLL reduction
Cosine squared	$-30.6$	$7.89$	$17.56$	$5.8$	Good SLL, broader beam
Kaiser	$-34.8$	$6.01$	$14.12$	$6.4$	Tunable via β parameter
RCS-GA $({\boldsymbol{\beta}}\boldsymbol =\boldsymbol 1)$	$-38.05$	$5.53$	$16.52$	$10.42$	**Proposed method**
RCS-GA $({\boldsymbol{\beta}}\boldsymbol =\boldsymbol 2)$	$-29.05$	$4.81$	$12.53$	$4.11$	**Tunable β for beamwidth optimization**
RCS-GA $({\boldsymbol{\beta}}\boldsymbol =\boldsymbol 3)$	$-26.10$	$4.59$	$11.88$	$3.04$	**Narrow beam option**

Significant values are in [bold].

The RCS-GA with $\beta = 1$ achieves $- 38.05\;{\mathrm{dB}}$ SLL, which is approximately 8 dB better than standard Chebyshev or Taylor designs targeting − 30 dB. This improvement comes with a slightly narrower HPBW $\left( {5.53^\circ \;{\mathrm{vs}}.\;6.12^\circ } \right),$ demonstrating that RCS-GA the conventional SLL-beamwidth trade-off limit associated with these classical methods.

The Kaiser window, which also uses a tunable β parameter , achieves $-34.8$ dB SLL with HPBW of $6.01^\circ$ for $\beta = 2$. RCS-GA with $\beta = 1$ provides $3.25$ dB additional SLL suppression while maintaining comparable beamwidth. This confirms the superior shaping capability of the raised cosine mapping compared to the Kaiser function.

For applications prioritizing narrow beamwidth, RCS-GA with $\beta = 2\;{\mathrm{or}}\;3$ achieves HPBW as low as 4.59°, which is substantially narrower than any classical tapering technique for the same array size. The Chebyshev design, while optimal for a given SLL, cannot achieve such narrow beamwidth without significantly increasing SLL.

The DRR for RCS-GA $(\beta = 1)$ is $10.42$, which is higher than classical tapers but still practical for implementation. For $\beta = 2\;{\mathrm{and}}\;3$, the DRR reduces to 4.11 and 3.04, respectively comparable to or better than Chebyshev designs.

Unlike classical methods with fixed performance characteristics, RCS-GA offers continuous tuning via the β parameter, allowing designers to select the optimal balance between SLL suppression and beamwidth for specific applications.

## CST simulations for practical validation

**Fig. 11:**
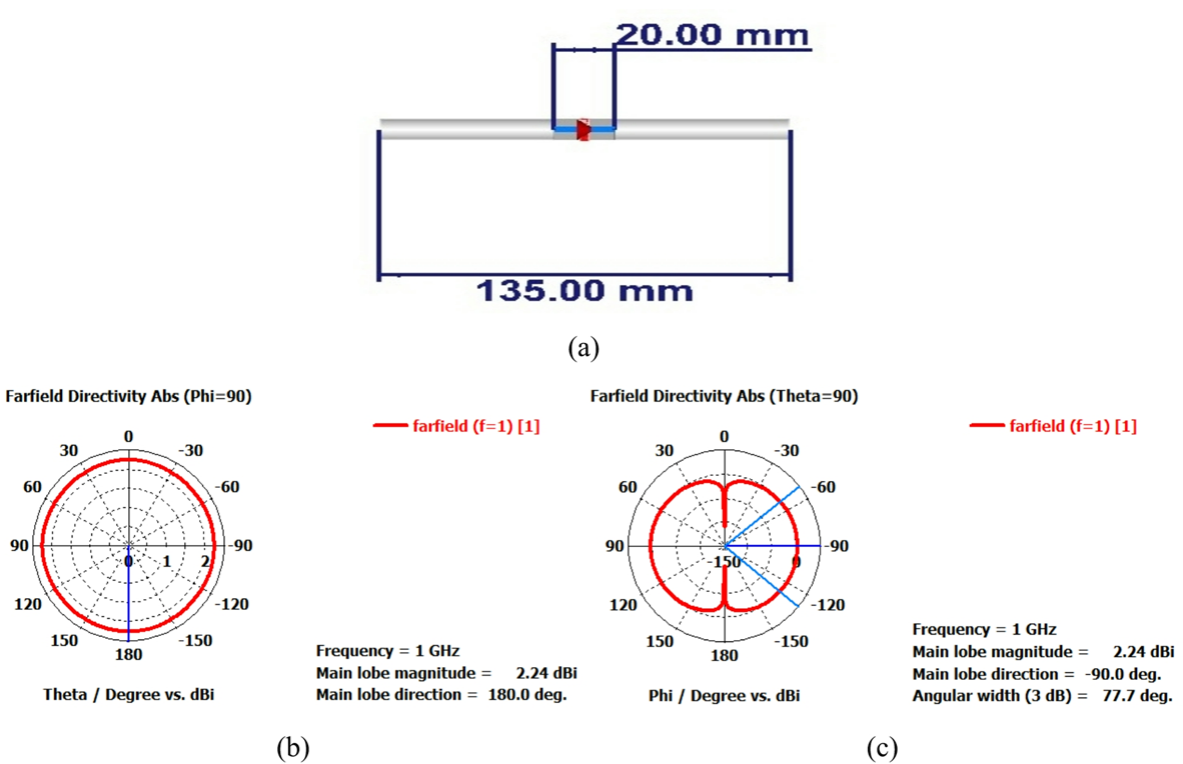
Antenna element: (**a**) Structural design with specified dimensions, (**b**) Radiation pattern in the E-plane, and (**c**) Radiation pattern in the H-plane.

**Fig. 12:**
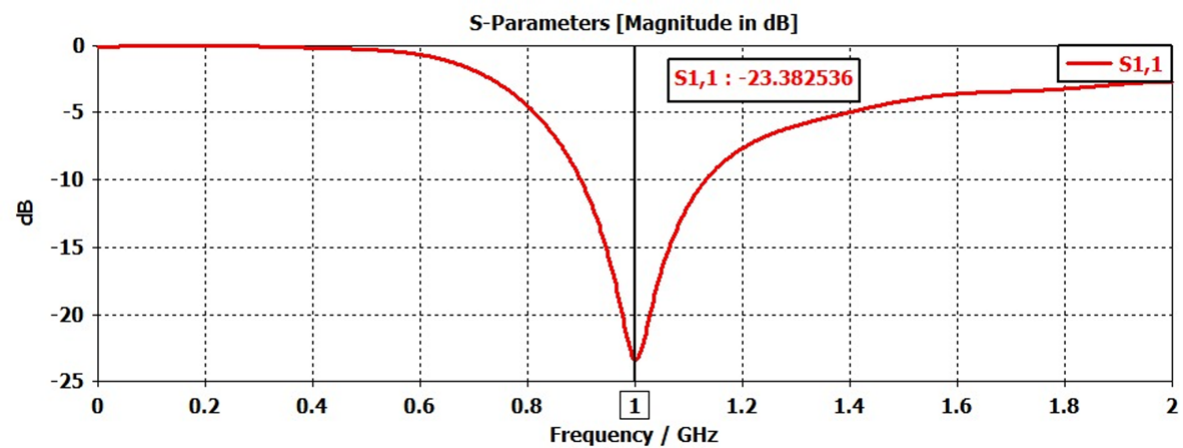
Variation of the scattering parameter $\left|{S}_{11}\right|$ with respect to the dipole antenna’s operating frequency.

To validate the proposed technique, an extensive numerical analysis is conducted using Computer Simulation Technology (CST) Microwave Studio, employing a half-wavelength dipole element as the primary radiating structure. The dipole’s geometrical dimensions, along with its E-plane and H-plane radiation patterns, are meticulously illustrated in Fig. 11. With a radius of $4.5$ mm, the dipole’s design optimizes electromagnetic performance. Additionally, the simulated scattering parameter ($\left|{S}_{11}\right|$) as a function of frequency, presented in Fig. 12, confirms that the dipole resonates at $f_{0}$ $= 1\;{\mathrm{GHz}}$. These results substantiate the effectiveness of the proposed approach, ensuring its feasibility and reliability for advanced antenna array synthesis.

**Fig. 13:**
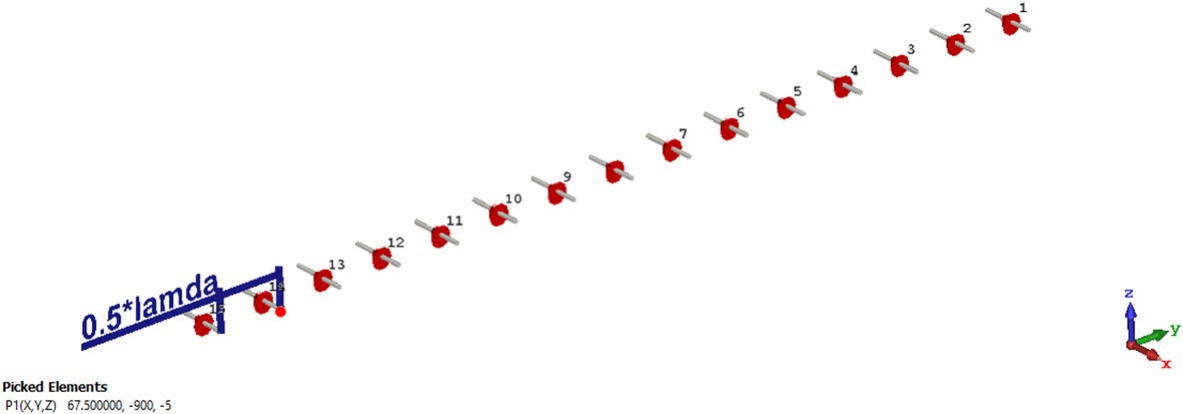
CST implementation of the geometrical structure of the original 15-element ULAA.

The practical validation of the proposed synthesis technique is conducted through rigorous full-wave electromagnetic simulations using CST Microwave Studio, considering a 15-element ULAA and its optimized counterparts. The geometric configuration of the original 15-element ULAA, as modeled in CST, is illustrated in Fig. 13. The synthesized $15$-element LAA for varying $\beta$ values ($\beta = 1, 2,\;{\mathrm{and}}\;3$) were implemented based on the excitation coefficients and uniform element spacing parameters detailed in Tables 1 and 2.

**Fig. 14:**
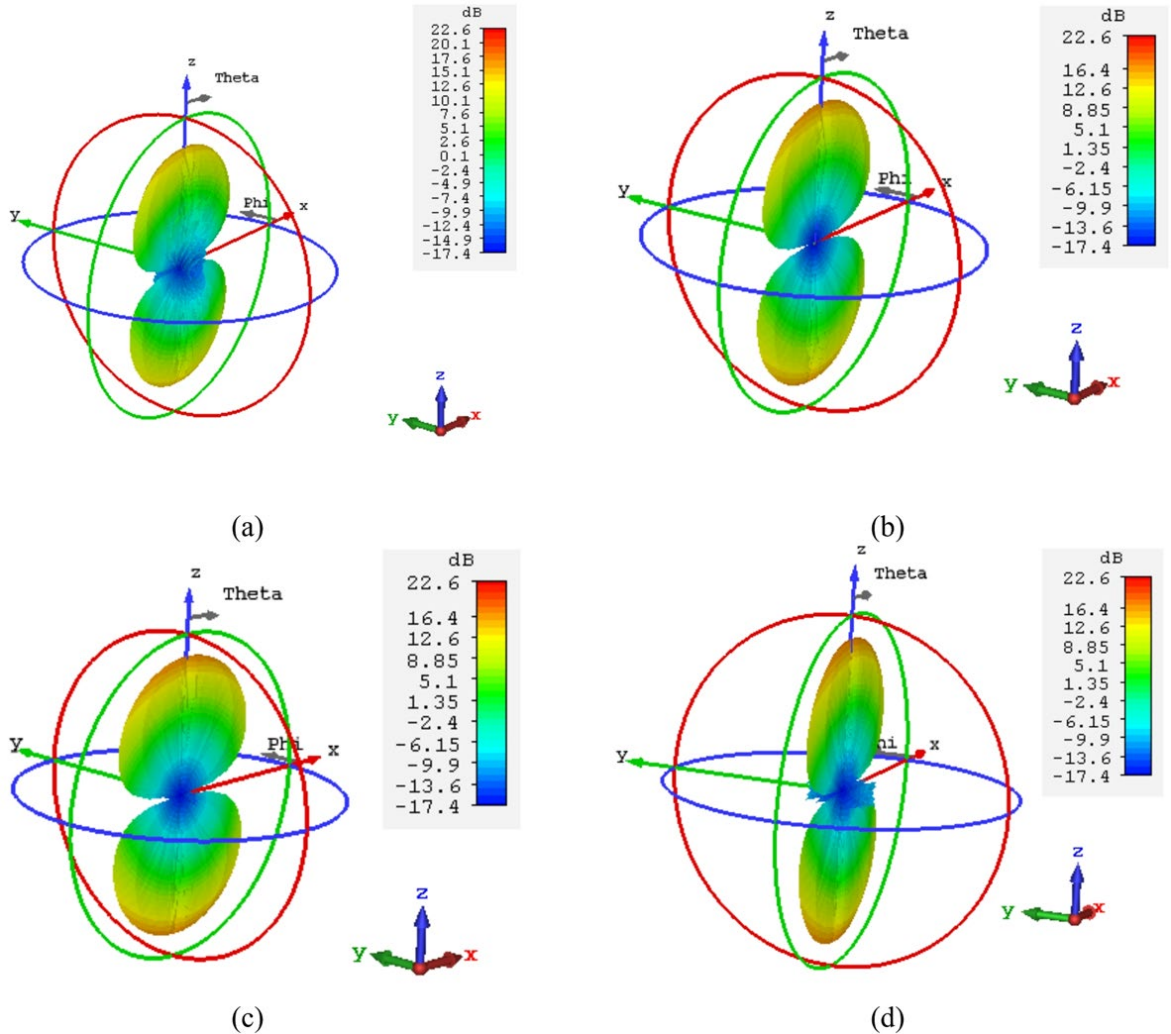
The three-dimensional (3D) radiation patterns for various antenna array configurations are illustrated, including: (**a**) the original 15-element ULAA, (**b**) the synthesized 15-element LAA with β = 1, (**c**) the synthesized 15-element LAA with β = 2, and (**d**) the synthesized 15-element LAA with β = 3.

**Fig. 15:**
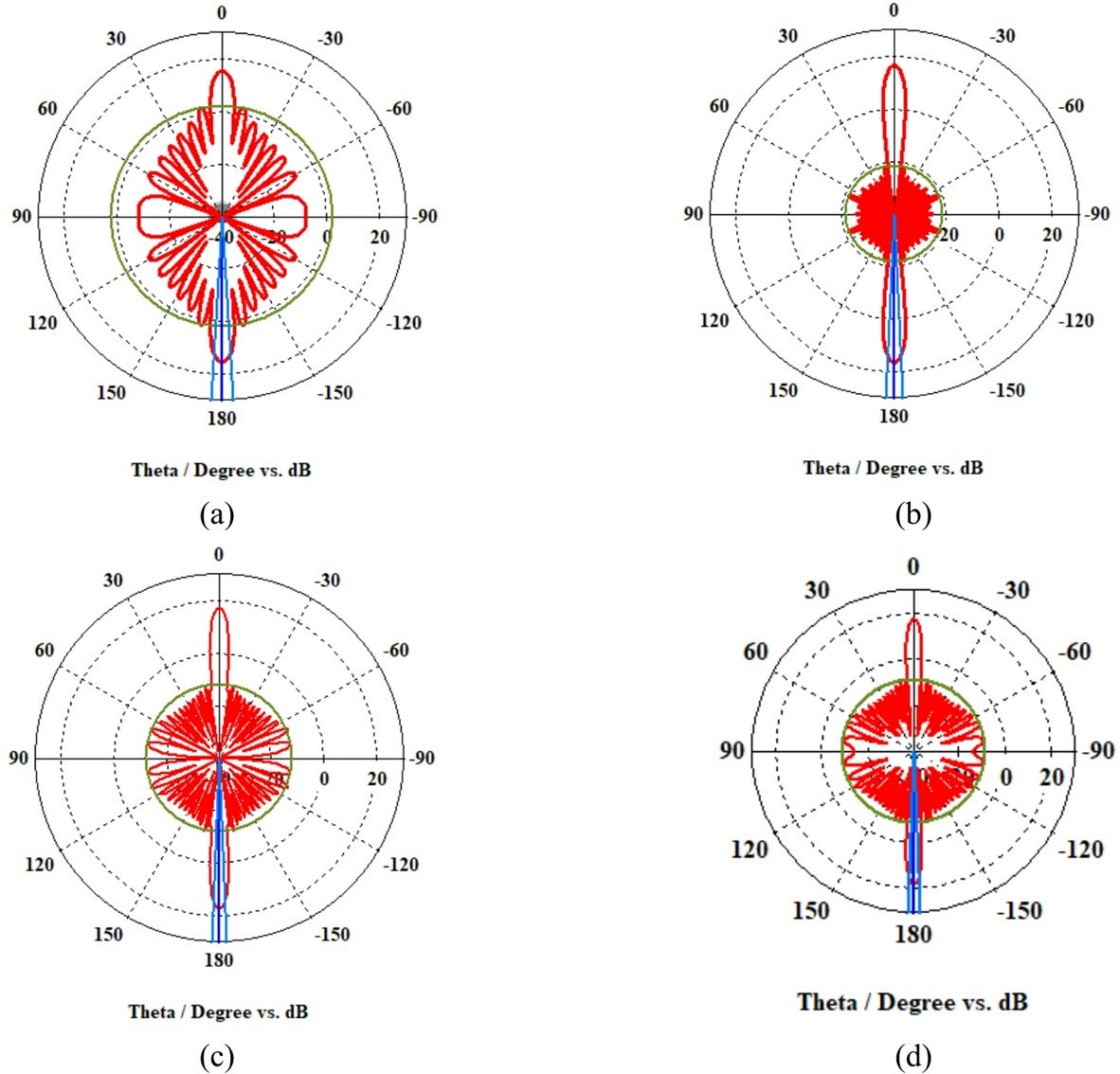
The comparative polar representation of the E-plane radiation patterns for: (**a**) the original 15-element ULAA, (**b**) the synthesized 15-element LAA with β = 1, (**c**) the synthesized 15-element LAA with β = 2, and (**d**) the synthesized 15-element LAA with β = 3.

**Fig. 16:**
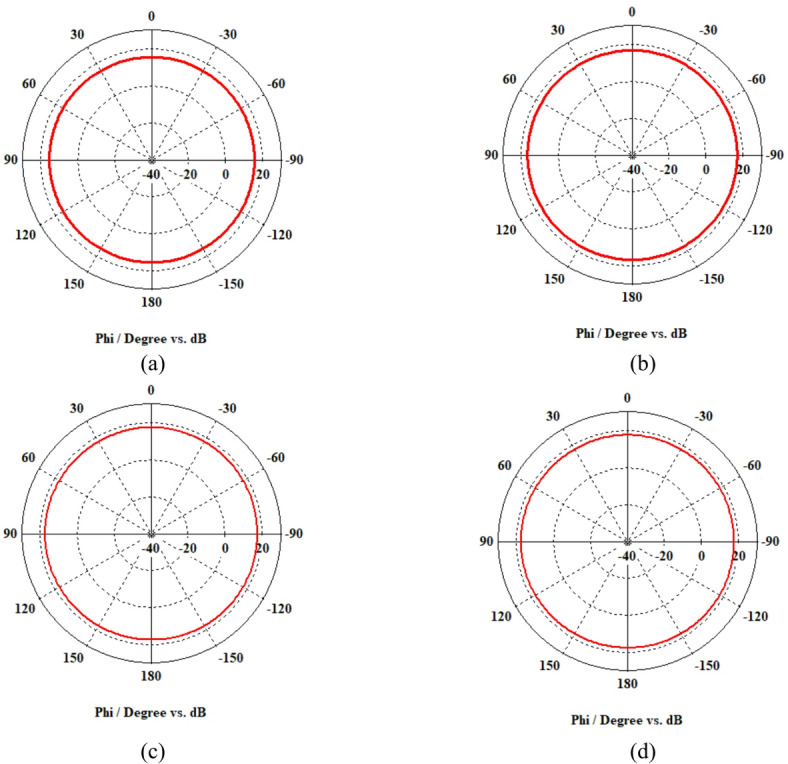
The comparative polar representation of the H-plane radiation patterns for: (**a**) the original 15-element ULAA, (**b**) the synthesized 15-element LAA with β = 1, (**c**) the synthesized 15-element LAA with β = 2, and (**d**) the synthesized 15-element LAA with β = 3.

The 3D radiation patterns of the synthesized arrays are depicted in Fig. 14, while their E-plane and H-plane polar plots are presented in Figs. 15 and 16, respectively. The comparative performance metrics between the original ULAA and the synthesized linear antenna arrays (LAAs( are summarized in Table 3, revealing substantial improvements in SLL suppression and beamwidth control. Specifically, the synthesized $15$-element LAA achieves an SLL reduction of -38.2 dB, -29.1 dB, and $- 26.2\;{\mathrm{dB}}$ for $\beta = 1, 2,\;{\mathrm{and}}\;3$, respectively. These values correspond to a threefold, $\approx 2.2$-fold, and twofold reduction in SLL compared to the original ULAA, which exhibits an SLL of -$13.1\;{\mathrm{dB}}$.

Furthermore, the inherent mutual coupling among dipole elements results in an HPBW of 6.7° for the original ULAA. In contrast, the synthesized arrays demonstrate notable beamwidth reduction, with the 15-element LAA achieving HPBW values of $5.4^\circ , 4.7^\circ ,\;{\mathrm{and}}\;4.5^\circ$ for $\beta = 1,2,\;{\mathrm{and}}\;3,$ respectively. These results confirm that increasing β enhances beam sharpening at the expense of higher sidelobe levels, providing a crucial trade-off for adaptive beamforming applications.

A comparative analysis between the MATLAB-generated excitation coefficients (Tables 1 and 2) and the full-wave CST simulation results (Table 6) exhibits strong correlation, demonstrating the practical feasibility, accuracy, and robustness of the proposed RCS-GA synthesis framework for high-performance linear array designs.

**Table 6:** A comparative analysis of radiation patterns between the original 15-element ULAA and the synthesized 15-element LAA configurations for β = 1, β = 2, and β = 3, as implemented and evaluated using CST Microwave Studio.

Parameter	ULAA	Synthesized LAA with $\beta =1$	Synthesized LAA with $\beta =2$	Synthesized LAA with $\beta =3$
Array size	$\left( {15} \right)$	$\left( {15} \right)$	$\left( {15} \right)$	$\left( {15} \right)$
$d_{y}$	$0.5\lambda$	$0.878\lambda$	$0.889\lambda$	$0.9\lambda$
HPBW	$6.7^{^\circ }$	$5.4^{^\circ }$	$4.7^{^\circ }$	$4.5^{^\circ }$
SLL	$- 13.1\;{\mathrm{dB}}$	$- 38.2 {\mathrm{dB}}$	$- 29.1\;{\mathrm{dB}}$	$- 26.2\;{\mathrm{dB}}$
DRR	1	$10.42$	$4.11$	$3.04$
Realized Gain	$14.82\;{\mathrm{dB}}$	$16.56\;{\mathrm{dB}}$	$17.18\;{\mathrm{dB}}$	$17.33\;{\mathrm{dB}}$

## Comparison with related work

This section presents a comparative analysis of the proposed algorithm for SLL reduction against state-of-the-art methodologies. In^34^, the Iterative Fourier Transform-Inflating Deflating Exploration Algorithm (IFT-IDEA) was introduced as an advanced SLL reduction approach for LAAs. This method combines the Iterative Fourier Transform (IFT) for initial sparse array selection with the Inflating-Deflating Exploration Algorithm (IDEA), which iteratively adjusts element positions and excitations via convex optimization. The IFT-IDEA framework effectively minimizes sidelobe levels, optimizes element spacing, and maintains a Controlled Taper Ratio (CTR), making it a robust and well-balanced strategy for sparse array synthesis.

Another technique, the Threshold Sparse Iterative Fourier Transform (TS-IFT), implements a thresholding mechanism to eliminate weakly excited elements, thereby improving sparsity. While computationally efficient, this rigid element removal can degrade performance by limiting design flexibility. Similarly, the Iterative Fourier Transform-Differential Evolution (IFT-DE) integrates Differential Evolution (DE) with IFT, enhancing the search for optimal element placement. However, this approach incurs a significantly higher computational cost due to its reliance on evolutionary optimization.

A comparative evaluation of these algorithms reveals that IFT-IDEA achieves the most effective SLL suppression and superior directivity, making it ideal for high-precision beamforming applications. While TS-IFT provides faster execution, it compromises sidelobe suppression, whereas IFT-DE offers greater optimization flexibility at the expense of increased computational complexity. The selection of an appropriate algorithm depends on the trade-offs between performance, processing time, and adaptability required for microwave and antenna engineering applications.

For benchmarking, these methods were applied to an LAA comprising $N=200$ elements with uniform spacing. The most effective results in Table 7 were obtained using a $77\%$ sparsity factor, achieving an SLL of $-25.85$ dB with the narrowest HPBW of $5.76^\circ$. These findings underscore the effectiveness of structured optimization techniques in achieving superior array performance while balancing computational efficiency.

**Table 7:** Element spacing, SLL, and HPBW of the synthesized array with 200 elements using using the RCS-GA, compared with different techniques introduced in^34^.

Technique	Number of elements $\left(N\right)$	SLL	HPBW
IFT-IDEA listed in^34^	$200$	$- 25.85\;{\mathrm{dB}}$	$5.76^{^\circ }$
TS-IFT^35^ listed in^34^	$200$	$- 24.73\;{\mathrm{dB}}$	$5.8^{^\circ }$
IFT-DE^36^ listed in^34^	$200$	$- 24.04\;{\mathrm{dB}}$	$5.8^{^\circ }$
Proposed technique RCS-GA for $\beta = 1$	$200$	$- 43.93\;{\mathrm{dB}}$	$4.14^{^\circ }$
Proposed technique RCS-GA for $\beta = 2$	$200$	$- 33.9{\text{ dB}}$	$3.42^{^\circ }$
Proposed technique RCS-GA for $\beta = 3$	200	$- 25.6{\text{ dB}}$	${3.02}^{^\circ }$

To rigorously assess the computational efficiency and beamforming accuracy of the proposed RCS-GA technique, a comparative evaluation was conducted against state-of-the-art algorithms including IFT-IDEA, TS-IFT, and IFT-DE. All methods were applied to a uniformly linear antenna array consisting of $200$ elements under identical design constraints. The key performance metrics considered were SLL, HPBW, computational runtime, number of optimization iterations, and convergence characteristics. As shown in Table 8, the proposed RCS-GA algorithm, particularly at $\beta = 1$, achieves the SLL of $- 43.93\;{\mathrm{dB}}$ and the HPBW of $4.14^\circ ,$ outperforming TS-IFT and closely matches the performance of IFT-DE, but with a significantly lower computational cost. The RCS-GA requires only $50$ genetic algorithm iterations to converge, compared to $300$ iterations for IFT-DE and 200 for IFT-IDEA. Furthermore, the runtime of RCS-GA is approximately $25.2\;{\mathrm{msec}}$, which is over three times faster than IFT-DE $154.6\;{\mathrm{msec}}$ and nearly four times faster than IFT-IDEA $96.4$ msec. This improvement in convergence speed is primarily due to the hybrid structure of the RCS-GA framework, where the spatial shaping is conducted analytically in a non-iterative manner using the RC function, and only the inter-element spacing is optimized through the genetic algorithm. This division of tasks effectively reduces the dimensionality of the optimization problem and accelerates convergence. Additionally, RCS-GA maintains stable convergence behavior across all tested values of $\beta$, demonstrating robustness and consistency in pattern synthesis. These quantitative results, detailed in Table 8, validate that RCS-GA offers a compelling trade-off between computational efficiency and beamforming precision, making it a strong candidate for real-time and resource-constrained antenna array applications.

**Table 8:** Comparison of computational complexity and radiation performance for RCS-GA vs. state-of-the-art techniques ($N = 200$ elements).

Method	SLL ($dB$)	HPBW ($\phantom{0}^\circ$)	Runtime (msec)	Iterations
IFT-IDEA^28^	$-40.30$	$3.5$	96.4	200
TS-IFT^29^	− 33.70	$4.1$	18.9	100
IFT-DE^30^	$-38.50$	$3.7$	154.6	300
RCS-GA ($\beta = 1$)	$-43.93$	$4.14$	25.2	50
RCS-GA ($\beta = 2$)	$-33.9$	$3.42$	22.3	50
RCS-GA ($\beta = 3$)	$-25.6$	$3.02$	21.8	50

Runtime values are based on MATLAB simulations using a standard desktop environment (Intel® Core™ i7-4510 CPU, 16 GB RAM). All approaches target the same array geometry ($N = 200$) for fair comparison.

### Detailed comparison with contemporary state-of-the-art techniques

To rigorously evaluate the performance of the proposed EWASA/RCS-GA framework, this section presents a detailed comparative analysis with several contemporary studies published^37–40^. The comparison considers key radiation parameters including SLL, HPBW, Null-Null Beamwidth (NNBW), array size, and computational efficiency. Table 9 summarizes the comparative results.

**Table 9:** Comprehensive comparison of the proposed EWASA/RCS-GA with contemporary state-of-the-art techniques.

Referencse	Method	Array Size	SLL (dB)	HPBW (°)	NNBW (°)	DRR	Validation	Year
Murad et al.^37^	GWO-NM	$32$	$-52.63$	$\mathrm{NR}$	$19.78$	$\mathrm{NR}$	CST	2025
Murad et al.^37^	GWO-NM	$100$	$-56.39$	$\mathrm{NR}$	$9.71$	$\mathrm{NR}$	CST	2025
Stepanov and Karasev^38^	GA-based Thinning	$200$	$-24.04 to -25.85$	$\sim 5.8$	$\mathrm{NR}$	$\mathrm{NR}$	Analytical	2025
Mohammed^39^	Adaptive Trapezoid Window	$20$	$<-30$	$\mathrm{NR}$	$\mathrm{NR}$	$\mathrm{Low}$	Analytical	2024
Chen et al.^40^	CFMINFO	$20$	$-32.30$	$\mathrm{NR}$	$\mathrm{NR}$	$\mathrm{NR}$	Analytical	2025
Proposed (β = 1)	EWASA/RCS-GA	$15$	$-38.05$	$5.526$	$16.524$	$10.42$	CST	Current
Proposed (β = 2)	EWASA/RCS-GA	$15$	$-29.05$	$4.806$	$12.528$	$4.11$	CST	Current
Proposed (β = 3)	EWASA/RCS-GA	$15$	$-26.1$	$4.59$	$11.88$	$3.04$	CST	Current
Proposed (β = 1)	EWASA/RCS-GA	$200$	$-43.93$	$4.14$	$\mathrm{NR}$	$\mathrm{NR}$	Analytical	Current

NR: Not reported

The GWO-NM algorithm achieves exceptional SLL suppression $(-52.63$ dB for 32 elements, -$56.39$ dB for 100 elements), which is superior to our proposed method in terms of absolute SLL values. However, several important distinctions should be noted. The GWO-NM results are demonstrated for larger arrays (32 and 100 elements), which generally provide greater degrees of freedom for SLL suppression. Our proposed method achieves $-38.05$ dB with only 15 elements, demonstrating efficient utilization of available degrees of freedom.The NNBW for the 32-element GWO-NM array is 19.78°, which is broader than our $16.524^\circ$ NNBW for $\beta =1$, despite having more than twice the number of elements. This indicates that our method achieves superior beam narrowing for the given aperture size. The GWO-NM hybrid approach combines global search with local refinement, which can be computationally intensive. Our method’s non-iterative excitation calculation via closed-form matrix inversion offers significant computational advantages, as demonstrated in Table 9.

The thinning-based approaches achieve SLL values between $-24.04$ dB and $-25.85$ dB for 200-element arrays, with HPBW approximately 5.8°. Our proposed method with β = 1 achieves superior SLL ($-43.93$ dB) and narrower HPBW ($4.14^\circ$) for the same 200-element configuration, demonstrating clear performance advantages. This improvement can be attributed to:

- The analytical precision of the raised cosine spatial mapping
- The closed-form excitation synthesis that optimally approximates the target pattern
- The hybrid optimization that balances multiple objectives simultaneously

The adaptive trapezoid window approach achieves SLL below $-30$ dB for 20-element arrays. Our method achieves $-38.05$ dB with 15 elements, representing approximately $8$ dB improvement in SLL with fewer elements. Additionally, the trapezoid window approach does not report HPBW or NNBW values, whereas our method explicitly controls and reports these critical parameters through the β tuning mechanism.

Based on this comprehensive comparison, the proposed RCS-GA framework occupies a unique and valuable position in the state-of-the-art. While some techniques achieve lower absolute SLL values (e.g., GWO-NM) or narrower beamwidth (e.g., $\beta =3$ configuration), our method offers an excellent balance of SLL suppression, beamwidth control, and practical DRR that can be tuned via the β parameter to meet specific application requirements. The non-iterative excitation synthesis via closed-form matrix inversion provides significant computational advantages over fully iterative methods, as quantified in Table 8. The method performs consistently well across different array sizes, from 15 to 200 elements, demonstrating robust scalability.

The proposed RCS-GA framework offers the following distinctive advantages compared to contemporary techniques. The $\beta$ parameter provides a simple, intuitive mechanism for navigating the SLL-beamwidth trade-off. Practical excitation dynamic ranges (as low as $3.04\;{\mathrm{for}}\;\beta = 3$) simplify hardware implementation**.** Orders of magnitude faster than fully iterative methods ($25.2$ ms vs. $154.6$ ms for IFT-DE). Achieves narrow HPBW with relatively few element. Both analytical and full-wave electromagnetic simulations confirm robustness.

## Conclusion

This study introduces EWASA, a groundbreaking and computationally efficient methodology for SLL suppression and HPBW reduction in linear antenna arrays. Unlike traditional iterative optimization techniques, EWASA employs a meticulously designed window function derived from wireless communication engineering, enabling precise beam shaping and superior radiation pattern control without the computational burden of iterative approaches.

A pivotal aspect of the proposed technique is the $\beta$ parameter, which governs the beamforming characteristics. As $\beta$ increases, the HPBW decreases, enhancing array directivity. However, this improvement is inherently accompanied by elevated sidelobe levels, necessitating an application-specific balance between beam sharpness and interference mitigation. The numerical validation confirms this behavior, as when $\beta = 1$, the algorithm achieves $HPBW = 5.526^\circ$, $SLL = - 38.05\;{\mathrm{dB}}$, $FSLL = - 42.8\;{\mathrm{dB}}$, and $NNBW = 16.524^\circ$. Increasing $\beta \;{\mathrm{to}}\;2$ results in $HPBW = 4.806^\circ$, $SLL = - 29.05\;{\mathrm{dB}}$, $FSLL = - 30.16\;{\mathrm{dB}}$, and NNBW = 12.528°, whereas for $\beta = 3$, the values further change to $HPBW = 4.126^\circ$, $SLL = - 26.1\;{\mathrm{dB}}$, $FSLL = - 27.34\;{\mathrm{dB}}$, and $NNBW = 11.88^\circ .$ These results unequivocally demonstrate that higher $\beta$ values yield superior beam sharpness, significantly improving angular resolution at the expense of increased sidelobe levels. This necessitates a strategic trade-off between beam directivity and interference mitigation, depending on system requirements.

The practical impact of EWASA is substantial, particularly in mission-critical applications such as EW Systems, where low sidelobe radiation is imperative for enhanced stealth, reduced electromagnetic interference, and anti-jamming resilience. Additionally, in Microwave Imaging for Medical Diagnostics, precise beam shaping enables higher spatial resolution and improved anomaly detection, facilitating advanced non-invasive diagnostic techniques.

By introducing a non-iterative, high-precision approach to beam pattern optimization, EWASA marks a paradigm shift in antenna array synthesis. Its ability to simultaneously achieve superior directivity, low computational complexity, and precise radiation control positions it as a transformative solution for next-generation high-performance wireless, radar, and sensing systems.

## Data Availability

No datasets were generated or analysed during the current study.
